# Targeted demethylation of the EphA7 promoter inhibits tumorigenesis via the SP1/DNMT1 and PI3K/AKT axes and improves the response to multiple therapies in cervical cancer

**DOI:** 10.1038/s41419-025-07512-4

**Published:** 2025-04-21

**Authors:** Wenfan Zhang, Jing Zhao, Xueting Fan, Shuang Chen, Rong Wang

**Affiliations:** 1 Department of Laboratory Medicine, School of Medical Technology, Tianjin Medical University, Tianjin, China; 2 Department of Gynecology and Obstetrics, Tianjin Medical University General Hospital, Tianjin, China

**Keywords:** Targeted therapies, Prognostic markers, Molecular biology

## Abstract

Aberrant methylation of the EphA7 promoter has been observed in cervical cancer (CC); however, its precise function and role in CC remain largely unknown. In this study, we investigated the role and molecular mechanisms of EphA7 promoter methylation in cervical carcinogenesis. First, our results indicated that the reactivation of EphA7 expression via a CRISPR demethylation tool (dCas9-Tet1) had antitumor effects. It restrained tumor proliferation and invasion while promoting apoptosis via the PI3K/AKT signaling pathway in both CaSki and SiHa cells. The upstream interacting factors were subsequently captured by CRISPR-mediated pull-down in situ, and the result revealed that SP1 and MAZ interacted with the promoter of EphA7. However, the perturbation results revealed that EphA7 expression was associated with SP1/DNMT1 but not MAZ. Furthermore, 17-β-estradiol (E2) can upregulate EphA7 expression through demethylation via the SP1/DNMT1 axis. A rescue experiment revealed that interference with SP1 expression could restore the effect of E2 on increasing the expression of EphA7 by upregulating estrogen receptor expression. In addition, EphA7 demethylation reduced the half-maximal inhibitory concentration (IC_50_) of cisplatin and paclitaxel. Pooled analysis revealed that EphA7 promoter hypermethylation was positively correlated with tumor purity but negatively correlated with immune cell infiltration, cytotoxic T lymphocyte (CTL) and immune checkpoint (IC) activity, and the expression of EphA7 was significantly positively correlated with tumor mutational burden (TMB), microsatellite instability (MSI) and the presence of single nucleotide variant (SNV) neoantigens, suggesting a better prognosis for patients with EphA7 promoter hypomethylation and high expression. Collectively, these findings indicate that targeted demethylation of the EphA7 promoter and restoration of endogenous EphA7 expression by dCas9-Tet1 are promising therapeutic approaches and are favorable for the prognosis of CC patients.

## Introduction

Cervical cancer (CC) ranks fourth in the incidence and mortality of cancer in women, with an estimated 660,000 new cases and 350,000 deaths worldwide in 2022 [1]. Recent statistics have shown that CC has moved up to become the third most common cause of cancer-related death among young women since 2019 in the United States [2]. A significant upward trend in mortality rates for CC patients from 2000–2018 in China was also observed [3]. Therefore, CC is considered a great threat to female health.

Despite significant advances in surgery, chemoradiotherapy and immunotherapies, drug resistance, recurrence, and metastasis of CC continue to significantly impact patient survival, with a 5-year survival rate of ~17.0% [4]. Currently, since PI3K signaling is one of the most frequently aberrantly activated pathways in cancer, the use of PI3K inhibitors to treat CC patients has been tentative. However, the response to PI3K inhibitors varies widely among different patients, in particular, infectious and immune-mediated toxicities limit their use at this stage [5]. Notably, further research into the molecular mechanisms of cervical carcinogenesis is essential for improving adjuvants and developing molecular-targeted compounds to accelerate precision medicine for CC.

EphA7 (Eph receptor A7) is a hypermethylation biomarker identified in CC in our previous study [6]. It belongs to the family of erythropoietin-producing hepatocellular carcinoma (Eph) receptors, which constitute the largest family of receptor tyrosine kinases (RTKs) [7]. Studies have shown that EphA7 is considered an indirect upstream regulator of the PI3K/AKT signaling pathway [8], contributing greatly to carcinogenesis, but its role is altered in different cancers [9].

In addition, since DNA methylation not only regulates gene expression but is also generally reversible, it holds enormous potential for directing clinical care [10]. Furthermore, the development of the clustered regularly interspaced short palindromic repeats (CRISPR) system of sequence-targeted technologies provides a bright future for manipulating DNA methylation and the interaction of transcription factors (TFs) [11], which provides additional insights into precise therapeutic approaches for CC. A previous study revealed that DNA methyltransferase 1 (DNMT1) is recruited by SP1 [12], and SP1 has been reported to be involved in cervical carcinogenesis [13, 14], which suggests that SP1 is a crucial TF that contributes to EphA7 methylation in CC.

Therefore, this study aimed to systematically investigate the function and mechanism of the epigenetic modulation of EphA7 in CC via CRISPR techniques, and we revealed that targeted demethylation of the EphA7 promoter inhibited CC progression via the PI3K/AKT pathway. In addition, we explored whether EphA7 promoter demethylation serves as an emerging prognostic and therapeutic target by examining its associations with estrogen, chemosensitivity, and immune checkpoints (ICs). Our findings provide new insights into biomarkers that integrate diagnosis with treatment for the targeted therapy of CC.

## Materials and methods

### Data collection and pooled analysis of EphA7

EphA7 promoter methylation and expression data were extracted from The Cancer Genome Atlas (TCGA; https://cancergenome.nih.gov/) and Gene Expression Omnibus (GEO; http://www.ncbi.nlm.nih.gov/geo/). The CC specimens collected from TCGA-CESC (Cervical squamous cell carcinoma and endocervical adenocarcinoma) cohort (*n* = 306) were included in the study, and according to the median of EphA7 promoter methylation level, the hypermethylation and hypomethylation groups were separated. GEO2R was used to identify differentially expressed genes (DEGs) related to EphA7 in GSE9750. Genes with adjusted *P* < 0.05 and |log2 FC | >2 were considered DEGs. The DAVID bioinformatics tool enables Kyoto Encyclopedia of Genes and Genomes (KEGG) enrichment analyses to predict the function of EphA7. TCGA plot was used to analyze the associations between EphA7 expression and the tumor mutational burden (TMB), microsatellite instability (MSI), and single-nucleotide variant (SNV) neoantigens in TCGA cancers.

### Cell lines and culture

The CC cell lines epidermoid carcinoma CaSki (RRID: CVCL_1100) and squamous cell carcinoma SiHa (RRID: CVCL_0032; Shanghai Zhongqiaoxinzhou Biotech, China), human embryonic kidney cells (HEK293T: RRID: CVCL_0063; Genetic Testing Biotechnology Corporation, Suzhou, China) and the normal cervical immortalized epithelial cell line H8 (BeiNa Culture Collection, China) were cultured in the appropriate media (DMEM, MEM or RPMI 1640). CaSki and SiHa cell lines were selected for this study because they are hypermethylation of EphA7 promoter and covered the important histological subtypes of CC. All cell lines were authenticated using short tandem repeat profiling within the last three years. All experiments were performed with mycoplasma-free cells. The cells were maintained in an environment supplemented with 10% FBS (BI, USA) and 1% antibiotics (penicillin/streptomycin) (Gibco, USA) at 37 °C with 5% CO_2_. For experimental treatments, cells were seeded at 70% confluence and exposed to 0.2 or 2 ng/ml 17-β estradiol (E2) (Solarbio) diluted in dimethyl sulfoxide (DMSO) for 48 h.

### Plasmid design and construction

As described in our previous study [6], single guide RNAs (sgRNAs) for targeting EphA7 promoter demethylation (referred to as “act-sgRNAs”) were designed via CRISPR-ERA (crispr-era.stanford.edu). In accordance with our previous optimization results [6], the combination of act-sgRNA1 + 3 was used to activate EphA7 in CaSki cells, and act-sgRNA1 + 2 was used in SiHa cells (Supplementary Table 1). Moreover, the top 5 potential off-target loci predicted by the COSMID web tool (https://crispr.bme.gatech.edu/) were selected. The primers used are listed in Supplementary Table 2. SgRNAs for CRISPR-based pull-down in situ (Cap-sgRNAs) were also designed to minimize off-target cleavage using the public tool CRISPR-ERA (Supplementary Table 1). The SP1 knockout sgRNA (KO-sgRNA) was designed as the reference described [15]. The annealed oligos were inserted into a modified pgRNA plasmid to create the sgRNA expression plasmids (Addgene #44248) with an AarI (Thermo, USA) site. For the knockdown of MAZ, target shRNA sequences (GATGCTGAGCTCGGCTTATAT) were subcloned and inserted into pLKO.1-shRNA lentivectors with EcoRI and AgeI (NEB, USA). The recombinant SP1 (NM_138473.3) overexpression plasmid was constructed by inserting the full-length cDNA into the pLVX-mCherry vector with XhoI and EcoRI (NEB, USA). The sequences of the sgRNAs and primers used for plasmid construction are detailed in Supplementary Table 1.

### Lentivirus production and stable cell line generation

Lentiviruses expressing Fuw-dCas9-Tet1CD (Addgene #84475), lenti-Cas9-Blast (Addgene #52962), sgRNAs, pLVX-SP1, and shMAZ were produced by transfecting HEK293T cells with those plasmids along with standard packaging vectors (psPAX2 and pMD2.G). The filtered Fuw-dCas9-Tet1CD lentivirus was concentrated via ultracentrifugation (System Biosciences, USA), as previously described [16].

Stable cell lines with CRISPR-captured FB-dCas9 and BirA were generated by transfecting pEF1a-FB-dCas9-puro (Addgene #100548) and pEF1a-BirA-V5-neo (Addgene #100547) into cells using polyethylenimine transfection reagent (Serochem). Stably transfected cells were treated with drug selection for 2 weeks or with a BD FACS Aria cell sorter on the basis of the selectable markers of the plasmids. The verification results for these stable cell lines are provided in Fig. S1A–C.

### Western blotting analysis

Western blotting analysis was performed as previously described [17]. HRP-labeled secondary antibodies were applied for 1 h at room temperature. The signals were detected using an enhanced chemiluminescence kit (Millipore, USA). GAPDH (Bioss) served as a loading control. The detailed information for the antibodies used for Western blotting is provided in Supplementary Table 3.

### Quantitative real-time PCR (qRT‒PCR)

The harvested cells were dissolved in TRIzol reagent (Invitrogen, USA), and the total mRNA was extracted according to the manufacturer’s protocol. qRT‒PCR assays were performed with SYBR Green (Vazyme) and a Stratagene Mx3005p instrument (Agilent, USA). The amplification parameters were as follows: 95 °C for 30 s, followed by 40 cycles of 95 °C for 10 s, and 60 °C for 30 s. The data were normalized to GAPDH expression, and the relative expression of target genes was calculated via the 2^−ΔΔCt^ method. The primers used for qRT‒PCR are available in Supplementary Table 2.

### Methylation-specific PCR (MSP)

The primers were designed via Methyl Primer Express v1.0 (Applied Biosystems, USA), and the primer sequences are listed in Supplementary Table 2. MSP was performed to assess the promoter methylation of EphA7, as previously described [6].

### Cell proliferation assay

For the detection of cell growth, 5,000 cells were seeded into each well of a 96-well plate and cultured for 0, 24, 48, or 72 h. Afterward, 50 μl of 1× MTT solution (KeyGEN BioTECH) was added and incubated for 4 h. Then, 150 μl of DMSO was added to the wells to dissolve the contents, and the optical density was determined by measuring the absorbance (490 nm) at the indicated time points.

### Transwell migration assays

For Transwell migration assays, 10,000 cells were suspended in 200 μl of serum-free medium and seeded into the upper chamber of each insert. Subsequently, 600 μl of culture medium containing 15% FBS was added to the lower chamber of a 24-well plate. After incubation at 37 °C, the cells that migrated were fixed, stained with 4% paraformaldehyde and 0.1% crystal violet, and rinsed with PBS. The cells attached to the chamber were observed under a microscope. The number of cells invading the Matrigel was considered to reflect the invasive capacity of the cells.

### Colony formation assays

For the clonogenicity analysis, 500 cells were seeded in six-well plates and cultured for 15 days. Afterward, the colonies were fixed with 4% paraformaldehyde for 30 min and stained with 0.05% crystal violet for 20 min.

### Measurement of E2 concentration

The supernatant was collected from SiHa-Tet1 and SiHa-Tet1 cells with sgRNAs, and the concentrations of E2 were measured via the Abbott Architect i2000SR system using chemiluminescence assays.

### Cytotoxicity assay

The cytotoxicity of cisplatin was assessed via MTT assays, as described previously [17]. CaSki and SiHa cells were treated with increasing concentrations of cisplatin or paclitaxel for 48 h. The half-maximal inhibitory concentration (IC_50_) was obtained from the optimized standard curve of the percentage of viable cells plotted against the extract concentration on the ordinate.

### In situ CRISPR-based pull-down assay

The in situ CRISPR-based pull-down assay for the EphA7 promoter was performed as previously described [11]. Affinity-purified chromatin was treated with RNaseA (Tiangen) and Protease K (G-Clone) and analyzed by qRT–PCR (Supplementary Table 2) to evaluate the sensitivity and specificity of cap-sgRNAs. TF detection was conducted after chemical cross-linking by formaldehyde and chromatin sonication. The proteins were separated via centrifugation and analyzed by western blotting.

### Statistical analysis

All image collection and data analyses were performed as double-blind experiments. Statistical analysis was performed using GraphPad Prism 8.0 (GraphPad Software, USA). Student’s *t* test was used to evaluate the significance of differences between two groups conforming to a normal distribution, whereas a Mann–Whitney *U* test was used for data not conforming to a normal distribution. One-way ANOVA was employed for multiple groups comparison. The associations between tumor purity, immune cell infiltration, chemosensitivity, immune-related genes, and methylation or expression levels were investigated by calculating Spearman’s rank correlation coefficients. The Wilcoxon test was performed to analyze the differences in expression between the complete response group and the group that did not achieve a complete response. Overall survival was compared between groups using Kaplan‒Meier curves and log-rank tests. At least three independent replicates were assessed for each of the in vitro experiments, and the pooled data are presented as the means ± standard errors. A *P* value < 0.05 was considered to indicate statistical significance (*P < 0.05, **P < 0.01, ***P < 0.001 and ns, not significant).

## Results

### Demethylation of the EphA7 promoter by the dCas9-Tet1 system inhibits CC progression and induces apoptosis

**Fig. 1:**
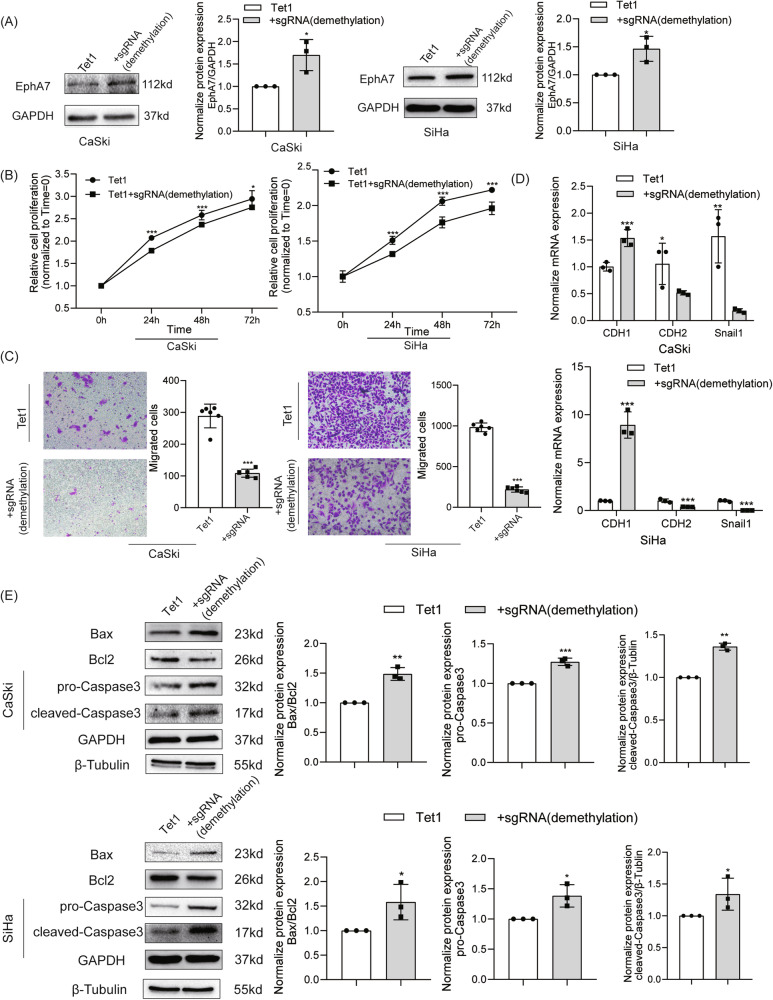
EphA7 demethylation via the dCas9-Tet1 system inhibited CC progression and induced apoptosis. **A** Western blotting was performed and revealed that EphA7 expression was upregulated in the dCas9-Tet1 with act-sgRNAs group (demethylation) compared with the dCas9-Tet1 group (control) in both CaSki and SiHa cells (*n* = 3). **B** Compared with the control, targeted demethylation of EphA7 effectively inhibited the proliferation of CaSki and SiHa cells, as determined via the MTT assay (*P* < 0.05) (*n* = 6). **C** Transwell assays revealed that EphA7 demethylation (*n* = 6) strongly reduced the number of migrated CC cells compared with that in the control group (*P* < 0.05). **D** The epithelial marker CDH1 (E-cadherin) was upregulated due to the demethylation of EphA7 (*n* = 3), whereas the mesenchymal markers CDH2 (N-cadherin) and Snail1 were downregulated in CaSki and SiHa cells. **E** Western blotting was used to verify that the demethylation of EphA7 increased the expression of Bax, downregulated the expression of Bcl-2 and promoted caspase-3 activity (*P* < 0.05) in CaSki and SiHa cells (*n* = 3). Error bars represent the means ± SDs, *P* values were calculated using two-tailed unpaired Student’s *t* tests (**A**–**E**). *P < 0.05, **P < 0.01, ***P < 0.001.

First, the methylation level of the EphA7 promoter was efficiently decreased by the dCas9-Tet1 tool (Fig. S2A) in our previous study [6]. The expression of EphA7 was restored (*P* < 0.05) at both the mRNA (Fig. S2B) and protein (Fig. 1A) levels in CaSki and SiHa cells. In addition, consistent with findings from other reports, the off-target effect of dCas9-Tet1 on DNA methylation was minimal [18]. Figure S2C, D shows no significant variation in the mRNA expression levels of the top five potential off-target genes predicted by the COSMID web tool in the demethylation groups of both CaSki and SiHa cells.

The enriched DEGs between the EphA7 high- and low-methylation groups were subsequently extracted from TCGA-CESCs, and the results indicated that EphA7 promoter methylation disrupted biological functions related to proliferation, metastasis, and apoptosis (Fig. S3A, B).

Our MTT (Fig. 1B) and colony formation (Fig. S3C) assays revealed that the demethylation of EphA7 profoundly blocked the proliferative activities of CaSki and SiHa cells, indicating that the demethylation of the EphA7 promoter inhibited the proliferation of CC cells. Fewer migrated CC cells were observed in the EphA7 promoter demethylation group than in the control groups (Fig. 1C).

The epithelial–mesenchymal transition (EMT) is a key step in tumor invasion and metastasis [19], and we further detected the expression levels of EMT-related genes in CC cells. As shown in Fig. 1D, the epithelial marker CDH1 was upregulated, whereas the mesenchymal markers CDH2 and Snail were downregulated in the EphA7-demethylation groups of both CaSki and SiHa cells. Overall, these findings imply that EphA7 demethylation might serve as an inhibitor of CC progression.

Subsequently, we found that the demethylation of EphA7 promoted cleaved-caspase-3 activity (*P* < 0.05), increased the expression of Bax, while downregulated the expression of Bcl-2 (*P* < 0.05) (Figs. 1E and S3D). The Bax/Bcl-2 ratio in CC cells was obviously increased (*P* < 0.05) in the EphA7-demethylation group (Fig. 1E).

### Demethylation of EphA7 inhibits CC progression via the PI3K/AKT pathway

**Fig. 2:**
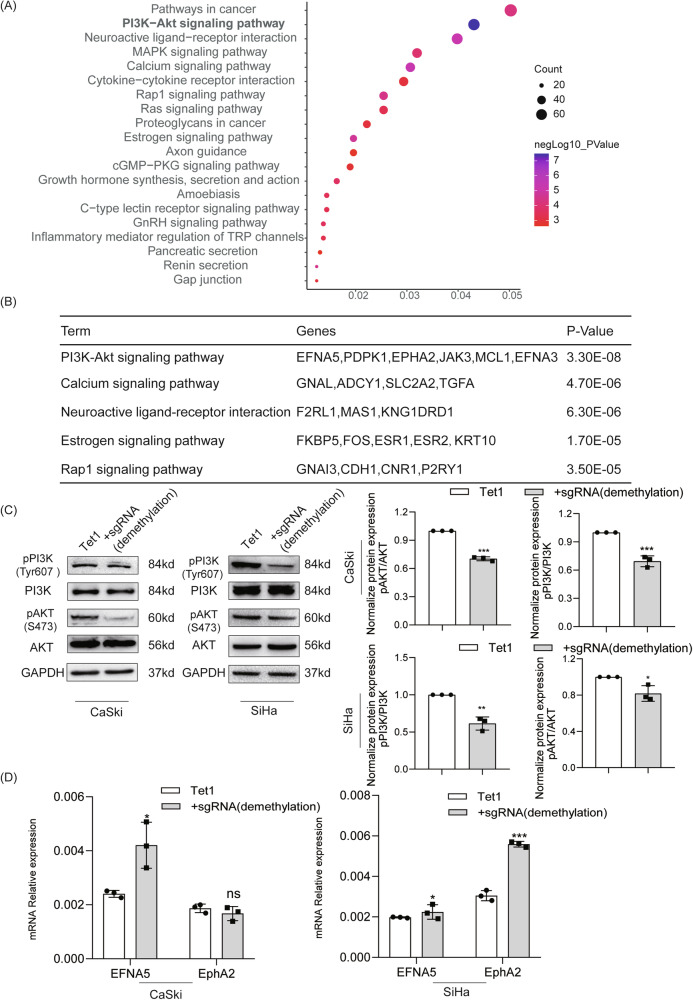
EphA7 demethylation inhibited CC progression via the PI3K/AKT signaling pathway. **A** These DEGs were obtained by comparing normal cervical samples with high EphA7 expression (*n* = 5) to CC samples with low EphA7 expression (*n* = 5) on the basis of EphA7 RNA-seq data (GSE9750). KEGG pathway enrichment analysis revealed that EphA7 significantly altered the PI3K/AKT signaling pathway. **B** Signaling pathways regulated by EphA7 according to the DAVID web tool. **C** The levels of the phosphorylated forms of PI3K and AKT were reduced, and the pPI3K/PI3K and pAKT/AKT ratios were decreased (*P* < 0.05) in the EphA7 demethylation group (*n* = 3). **D** The expression of EFNA5 and EphA2 was increased in the EphA7 demethylation group (*n* = 3). Error bars represent the means ± SDs, *P* values were calculated using two-tailed unpaired Student’s *t* tests (**C**, **D**). *P < 0.05,  **P < 0.01, ***P < 0.001 and ns, not significant.

We extracted RNA-seq data from GSE9750 and subsequently performed a KEGG pathway enrichment analysis on the DEGs (Fig. S4A). The results revealed that EphA7 significantly altered the PI3K/AKT signaling pathway (Fig. 2A, B).

As depicted in Fig. 2C, the pPI3K/PI3K and pAKT/AKT ratios were decreased in the EphA7 demethylation group (*P* < 0.05). Furthermore, the EphA7 protein was found to directly bind to EFNA5 and EphA2 according to the STRING analysis (Fig. S4B). In addition, single-cell RNA sequencing revealed a positive association between EphA7 expression and EFNA5/EphA2 expression in human cells using Cellxgene tools (Fig. S4C). Consistent with these findings, qRT‒PCR confirmed that the expression of EFNA5 and EphA2 was upregulated in the EphA7 demethylation group (*P* < 0.05) (Fig. 2D).

### SP1 regulates the methylation of EphA7 by interfering with DNMT1

**Fig. 3:**
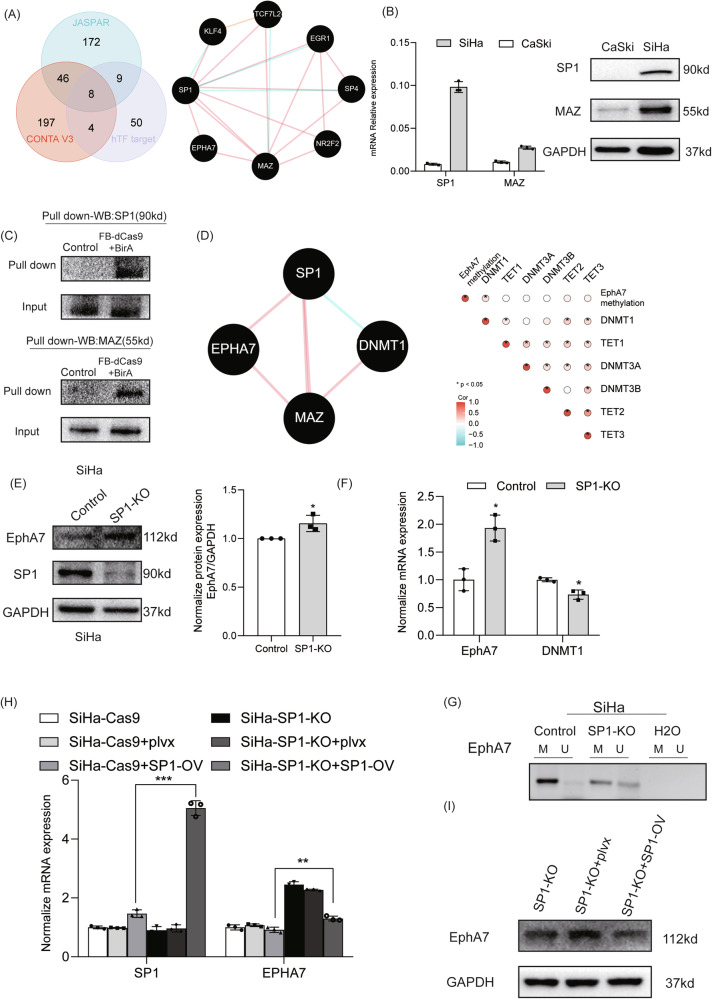
SP1 regulated the methylation of EphA7 by interfering with DNMT1. **A** The JASPAR (http://jaspar.genereg.net/), CONTA V3 (http://bioit2.irc.ugent. be/contra/v3), and hTFtatget (http://bioinfo.life.hust.edu.cn/ hTFtarget) tools were integrated to predict transcription factor binding in the promoter of EphA7. SP1 and MAZ were found to directly interact with EphA7 on the basis of the Pathway Commons (https://www.pathwaycommons.org). **B** The mRNA and protein expression analysis showed that total SP1 and MAZ were significantly expressed in SiHa cells. **C** The in situ pull-down results verified that SP1 and MAZ were located in the promoter region of EphA7 in SiHa cells. **D** Pathway commons tools indicating that SP1 and MAZ directly interact with EphA7 and DNMT1. Promoter methylation of EphA7 was associated with DNMT1 (*P* < 0.05) in TCGA-CESCs (*n* = 306). **E** Western blotting was performed to demonstrate that SP1 was silenced in SiHa-SP1 knockout cells (SiHa-SP1-KO) and that the protein expression levels of EphA7 were increased in SP1-KO cells (*n* = 3). **F** The mRNA expression levels of EphA7 were increased, and the expression of DNMT1 was decreased in SP1-KO cells (*n* = 3). **G** Knockout of SP1 markedly reduced the methylation level of EphA7 in SiHa cells. **H**, **I** The results of rescue assays showed that SP1 overexpression reversed the changes in the mRNA (*P* < 0.05) and protein expression of EphA7 in the SP1-KO group. Error bars represent the means ± SDs, *P* values were calculated using two-tailed unpaired Student’s *t* tests (**E**–**G**) and Spearman’s analysis (**D**). *P < 0.05, **P < 0.01,  ***P < 0.001.

Eight predicted binding sites were identified in the EphA7 promoter region through an overlapping TF database, including JASPAR, hTFtarget, and ConTra v3 (Fig. 3A). SP1 and MAZ were found to directly interact with EphA7 via the Pathway Commons integrated tool (Fig. 3A). The predicted binding sites are listed in Supplementary Table 4, and the sites of capture-sgRNAs are shown in Fig. S5A. Although the EphA7 promoter was hypermethylated in SiHa and CaSki cells (Fig. S5B), as shown in Figs. 3B and S5C, total SP1 and MAZ were significantly more highly expressed in SiHa cells than in CaSki cells. Furthermore, consistent with the experimental results, we also found that SP1 was indeed expressed at lower levels in adenocarcinoma (ADC) (Fig. S5D) than in squamous cell carcinoma (SCC) from the TCGA. Next, in situ CRISPR pull-down techniques were used to elucidate the upstream regulatory mechanism of EphA7. The results verified that Cap-sgRNA1-4 effectively enriched fragments of the EphA7 promoter in SiHa cells (*P* < 0.05) (Fig. S5E) and confirmed that SP1 and MAZ were indeed located in the EphA7 promoter region in SiHa cells (Fig. 3C). As controls of in situ pull-down technics, we performed parallel experiments in H8 cells of and the results showed that SP1 and MAZ were enriched as well (Fig. S5E, F).

By applying the Pathway Commons, SP1 and MAZ were discovered to directly interact with EphA7 and DNMT1 (Fig. 3D). In addition, EphA7 promoter methylation was positively associated with DNMT1 expression in TCGA-CESCs (Fig. 3D). Knockout of SP1 significantly increased the expression of EphA7 (Fig. 3E) and downregulated the expression of DNMT1 (*P* < 0.05) (Fig. 3F). We also found that the level of EphA7 methylation in SiHa cells was reduced in the SP1 knockout group (Fig. 3G). However, MAZ knockout did not affect the expression of DNMT1(Fig. S5G).

Notably, both the protein and mRNA expression levels of EphA7 increased in SiHa-SP1-KO cells (Fig. 3E, F). Conversely, the overexpression of SP1 decreased EphA7 expression while upregulating DNMT1 (Fig. S5H) in SiHa cells.

We performed a rescue experiment using an SP1 overexpression plasmid in SiHa-SP1-KO cells to further confirm the direct association between SP1 and EphA7. SP1 overexpression reversed the changes in EphA7 mRNA and protein expression in the SP1-KO group (Fig. 3H, I), indicating that EphA7 expression is directly regulated by SP1 in SiHa cells.

### SP1 interacts with the ER to regulate EphA7 methylation

**Fig. 4:**
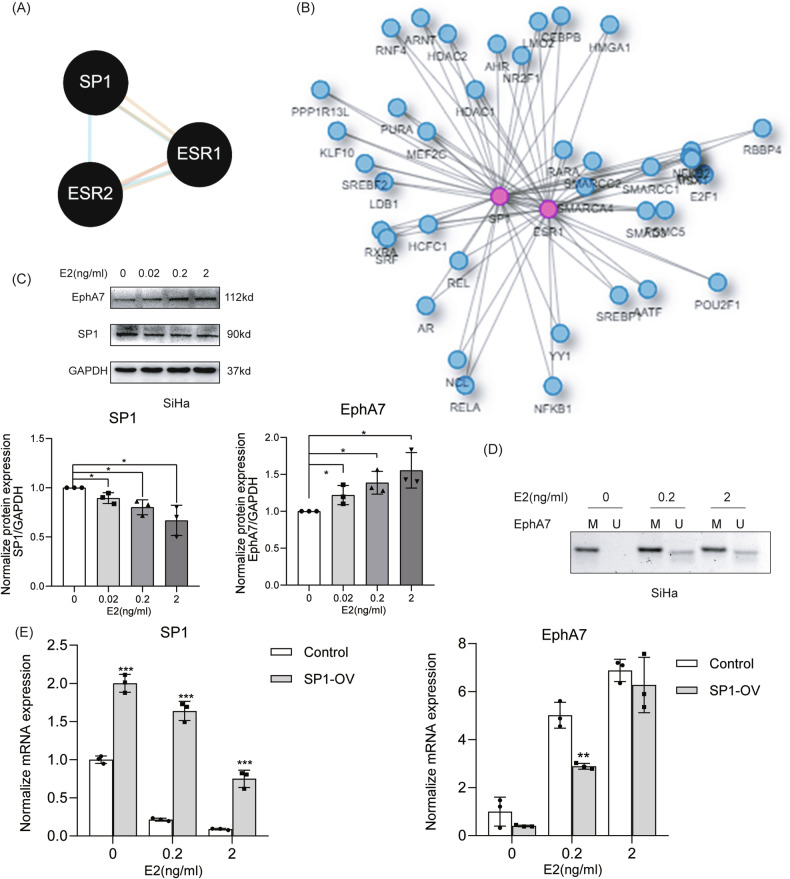
SP1 interacts with the ER to regulate EphA7 methylation. **A** The Pathway Commons web tool demonstrated that SP1 interacted with ESR1 and ESR2. **B** According to Integrative Interactome Database (http://ophid.utoronto.ca/iid), SP1 was found to interact with ESR1 in human uterine tissues. **C** The protein level of SP1 was obviously reduced (*P* < 0.05), and the protein level of EphA7 gradually increased in a dose-dependent manner with increasing concentration (*P* < 0.05) (*n* = 3). **D** The methylation level of EphA7 was obviously reduced in E2-treated SiHa cells. **E** The increase in EphA7 levels due to E2 treatment was partly reversed by SP1 overexpression, as determined by a rescue assay (*n* = 3). Error bars represent the means ± SDs, *P* values were calculated using two-tailed unpaired Student’s *t* tests (**E**), and one-way ANOVA with Tukey’s multiple comparisons test (**C**). *P < 0.05, **P < 0.01, ***P < 0.001.

As depicted in Fig. 2B, KEGG pathway enrichment analysis revealed that EphA7 obviously altered the estrogen signaling pathway (*P* < 0.05). According to Pathway Commons, SP1 interacted with ESR1 and ESR2 (Fig. 4A). Furthermore, the Integrative Interactome Database revealed that SP1 interacts with ESR1 in human uterine tissues (Fig. 4B).

Following the treatment of SiHa cells with E2, the mRNA expression of ESR1 and ESR2 generally increased (Fig. S6). Following the treatment of SiHa cells with E2, the expression of SP1 (Fig. 4C) and the level of EphA7 methylation were obviously reduced (*P* < 0.05) (Fig. 4D). Furthermore, both the mRNA and protein levels of EphA7 gradually increased in a dose-dependent manner after treatment with increasing E2 concentrations (Fig. 4C, E). In addition, as shown in Fig. 4E, the increased levels of EphA7 resulting from E2 treatment were partially reversed by SP1 overexpression. Moreover, we detected that much less E2 was produced by SiHa-Tet1 or SiHa-Tet1 cells with sgRNAs, which demonstrates that the regulation of EphA7 is not affected by endogenous E2(Supplementary Table 5).

### Demethylation of EphA7 is associated with a favorable response to chemotherapy in CC

**Fig. 5:**
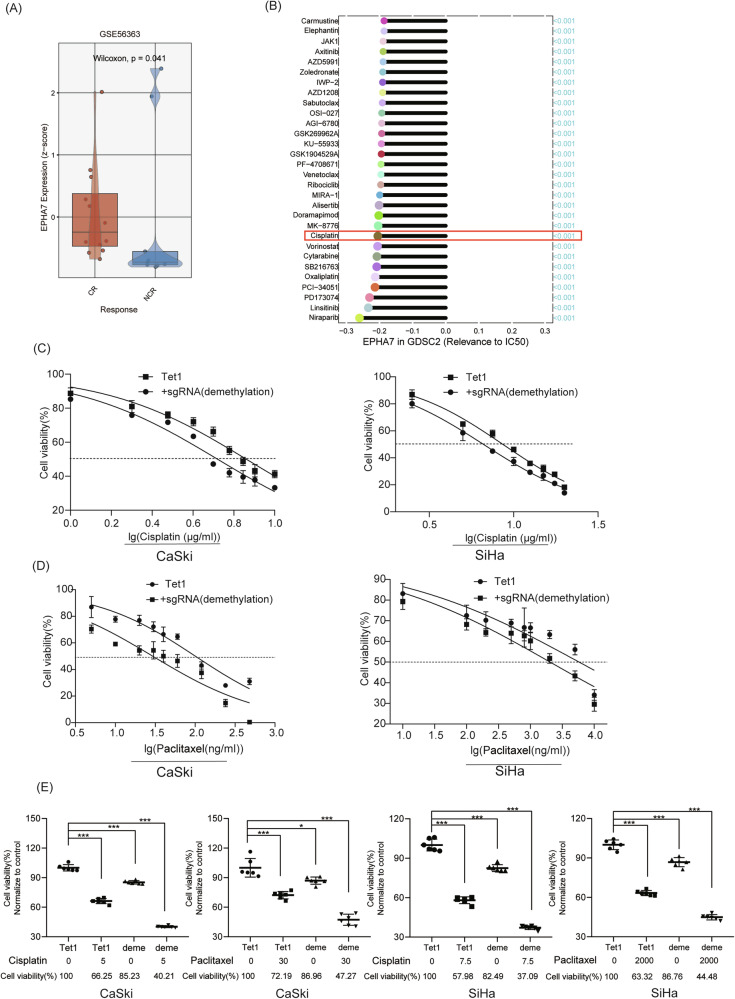
The demethylation of EphA7 is associated with a favorable response to chemotherapy in CC patients. **A** The results showed that the expression of EphA7 was greater in the CR group than in the NCR group (*P* < 0.05) through the BEST web tool in the GSE56363 dataset. **B** Analysis of the association between EphA7 expression and sensitivity to chemotherapeutic drugs via Guangreshengwu (https://grswsci.top/analyze). **C** EphA7 demethylation appreciably reduced the half-maximal IC_50_ of cisplatin in CaSki and SiHa cells compared with that in the control group (*n* = 6). **D** The IC_50_ of paclitaxel was lower in the experimental group than in the demethylation group (*n* = 6). **E** CRISPR-targeted demethylation of EphA7 worked synergistically with cisplatin/paclitaxel, significantly increasing cisplatin/paclitaxel susceptibility. Error bars represent the mean ± SD, *P* values were calculated using two-tailed unpaired Student’s *t* tests (**E**). *P < 0.05. *P < 0.05, **P < 0.01, ***P < 0.001.

Figure 5A shows greater EphA7 expression in the complete response (CR) group than in the noncomplete response (NCR) group (*P* < 0.05) in the GSE56363 dataset. In addition, the association between EphA7 expression and sensitivity to therapeutic drugs revealed a negative correlation with IC_50_ values of cisplatin (*P* < 0.05) (Fig. 5B).

Consistent with these results, our findings shown in Fig. 5C, D indicate that EphA7 demethylation significantly reduced the IC_50_ values of cisplatin and paclitaxel in CaSki and SiHa cells compared with those in the control group. As shown in Fig. 5E, CRISPR-targeted demethylation of EphA7 worked synergistically with cisplatin/paclitaxel, significantly increasing cisplatin/paclitaxel susceptibility (*P* < 0.05). This finding suggested that EphA7 demethylation effectively improves the chemosensitivity of CC cells and that the demethylation of EphA7 appears to be associated with a favorable response of CC to chemotherapy.

### EphA7 methylation is a potential immunotherapy biomarker involving the tumor microenvironment (TME) in CC

**Fig. 6:**
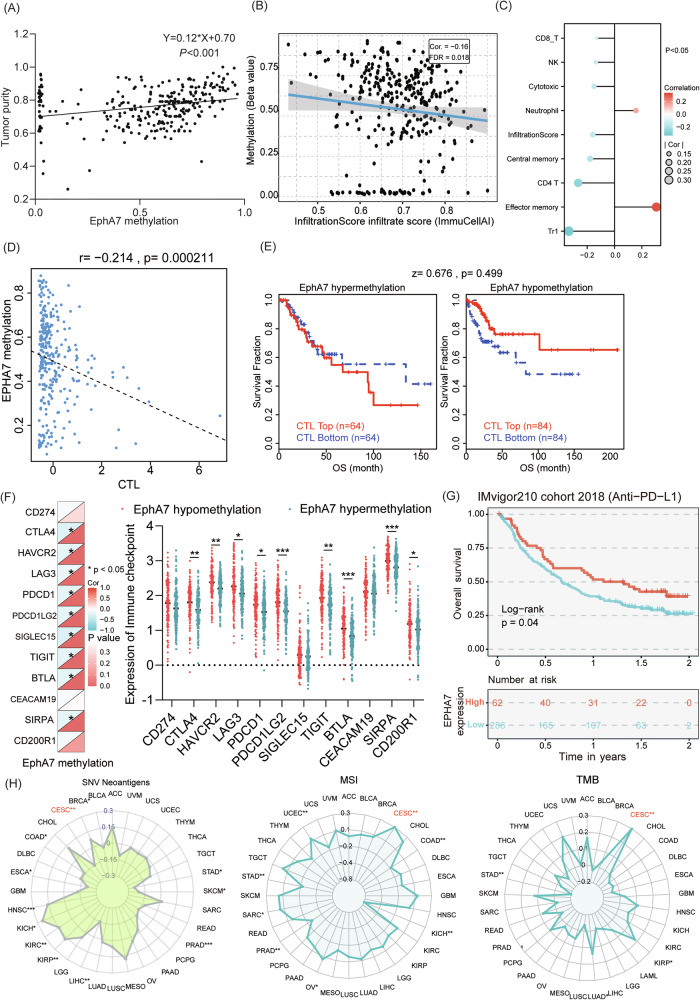
EphA7 is a potential immune therapy biomarker involving the TME in CC. **A** Tumor purity was positively correlated with the extent of EphA7 methylation (*cor* = 0.115, *P* < 0.05) in CESCs according to the Linkomics tool (http://www.linkedomics.org/login.php) in CESCs (*n* = 306). **B** The results showed that the infiltration score of CESCs (*n* = 306) negatively correlated with EphA7 methylation (*cor* = −0.16, *P* < 0.05) via GSCA (https://guolab.wchscu.cn/ GSCA/#/). **C** Spearman correlation analyses showing the association between the methylation levels of EphA7 and immune cell infiltration via GSCA. **D**, **E** Promoter methylation of EphA7 was negatively correlated with CTLs (*cor* = −0.214), and the population with low EphA7 methylation had a better prognosis in the CTL top group on the basis of the TIDE algorithm (http://tide.dfci.harvard.edu/). **F** Comparative analyses of IC expression profiles across EphA7 hypo/hypermethylation cohorts in TCGA-CESCs (*n* = 306). The upper triangle represents *P* value. The lower triangle in the heatmap represents the correlation coefficient. Red indicates a positive correlation, and blue indicates a negative correlation. **G** Patients with anti-PD-L1 antibodies had a better prognosis in the group with high EphA7 expression (*P* < 0.05) by the web tools BEST (https://rookieutopia.com/app_direct/BEST/). **H** EphA7 expression was significantly positively associated with SNV neoantigens, MSI status, and TMB (*P* < 0.05). The abbreviations and full names are listed in Supplementary Table 6. *P* values were calculated using two-tailed unpaired Student’s *t* tests (**F**) and Spearman’s analysis (**A**, **B**, **D**, **H**). *P < 0.05,**P < 0.01, ***P < 0.001.

Tumor purity is one way to determine the efficacy of immunotherapy, and CC patients with low tumor purity tend to have a better prognosis [20]. As shown in Fig. 6A, the level of EphA7 hypermethylation was positively correlated with the tumor purity of CESCs (*cor* = 0.115, *P* < 0.05).

The immune infiltration score can predict the prognosis and efficacy of immunotherapy for multiple cancers [21–23]. GSCA revealed that EphA7 hypermethylation was negatively correlated with the infiltration score (*cor* = -0.16, *P* < 0.05) and infiltration of 8 immune cell types, including type 1 regulatory T (Tr1) cells, CD4 + T cells, central memory cells, cytotoxic cells, NK cells, and CD8 + T cells (Fig. 6B, D). Conversely, a positive association between EphA7 hypermethylation and the infiltration of effector memory and neutrophil cells was observed (*P* < 0.05) (Fig. 6C). Notably, Kaplan‒Meier curves showed that high cytotoxic T lymphocyte (CTL) levels were associated with prolonged survival in the EphA7 hypomethylation group (Fig. 6E). The results revealed that patients with CC in the EphA7 hypomethylation group had a favorable response to immunotherapy.

Studies have shown that patients with high expression of ICs are more likely to respond to immunotherapy [24]. We found that EphA7 promoter methylation was negatively correlated with the expression of IC genes, and the EphA7 hypomethylation group presented higher mRNA expression levels of IC genes, including CTLA4, HAVCR2, LAG3, PDCD1, PDCD1LG2, TIGT, BTLA, SIRPA, and CD200R1 (*P* < 0.05) (Fig. 6F). Moreover, EphA7 promoter methylation was negatively correlated with the expression of chemokines, chemokine receptors, and major histocompatibility complex (MHC) genes (Fig. S7), indicating that the EphA7 hypomethylation group may be more sensitive to immune checkpoint blockade (ICB) therapy.

Finally, we observed that patients in the high EphA7 expression group who received anti-PD-L1 treatment had a better prognosis (*P* < 0.05) (Fig. 6G). EphA7 expression was positively correlated with TMB, MSI, and the presence of SNV neoantigens in CESC patients (*P* < 0.05) according to the TCGAplot and Guangreshengwu web tool (Fig. 6H). These findings may significantly contribute to the development of targeted immunotherapy and improved treatment strategies for CC.

## Discussion

In this study, we first demonstrated that EphA7 functions as a tumor suppressor in CC via the PI3K/AKT signaling pathway by activating endogenous EphA7 expression via demethylation. We also revealed that the demethylation of EphA7 is associated with a favorable response to antitumor therapy, which indicates that EphA7 methylation may be an emerging prognostic marker and therapeutic target.

Previous research revealed that DNA methylation occurs at different steps of tumorigenesis, and given that epigenetic dysregulation is reversible, epigenetic modifications have been confirmed as potential targets for cancer therapy [25]. Owing to the nonspecific nature and wide range of side effects of epi-drugs [26], the CRISPR system for sequence-targeted demethylation has a broad future for manipulating DNA methylation and regulating endogenous expression in a targeted manner [27], as evidenced by EphA7 demethylation in our previous and current studies.

Numerous studies have shown that the PI3K/AKT pathway is abnormally activated in CC [28–30]. As an upstream factor, EphA7 interferes with PTEN/PI3K/AKT signaling to regulate cell apoptosis in prostate tumors [31] and laryngeal squamous cell carcinoma [32]; however, little information on the role of EphA7 in CC has been obtained to date. Therefore, both the KEGG pathway enrichment analysis and the experimental findings confirmed that the demethylation of EphA7 in CC impairs the PI3K/AKT signaling pathway (Fig. 2C).

In addition, Dr Xu and colleagues have harnessed CRISPR-dCas9 technology by coupling it with FB/BirA to construct an in situ pull-down tool [18], which allows us to further explore the interacting factors upstream of the target gene. Previous studies have shown that SP1 positively regulates the transcription of DNMT1, increasing the risk of hypermethylation of gene promoters [33]. By combining bioinformatics information and the CRISPR-based in situ pull-down tool, we also successfully identified SP1 as a crucial factor that regulates the methylation of EphA7 by interfering with DNMT1 (Fig. 3G).

Estrogen, a key hormone, plays a multifaceted role in CC. It has been found to decrease mitochondrial membrane permeability, promote the Warburg effect, and increase antiapoptotic protein levels, which favor the metabolic adaptation of CC cell lines and cervical squamous carcinoma survival [34, 35]. Riera-Leal A et al. reported that cotreatment with E2 enhances chemotherapeutic agent sensitivity in SiHa cells (HPV positive) but reduces apoptosis in C33A cells (HPV negative) [36]. Interestingly, Young MJ et al. further revealed that SP1 could interact with the estrogen receptor ER to coregulate gene expression [37]. Our study discovered that despite the regulation of EphA7, substantially less E2 is secreted from SiHa cells, but exogenous E2 impairs SP1 function, leading to reduced EphA7 methylation and the reversal of EphA7 expression. The present study provides strong evidence for the efficacy of estrogen replacement therapy in patients with cervical squamous cell carcinoma.

Cisplatin monotherapy and combination regimens are standard chemotherapeutic approaches for treating CC [38]. Unfortunately, drug resistance compromises the efficacy of chemotherapy. Therefore, researchers are actively exploring strategies to increase cancer cell sensitivity to drugs and improve the survival rate of CC patients. A previous study tested therapies combining cisplatin with other cancer drugs in cancer cell lines, but few anticancer drugs have been found to exert synergistic effects [39]. Sun Hee Lee et al. discovered that DNA methylation inhibitors such as 5-aza-2-deoxycytidine can work synergistically with cisplatin to increase the efficacy of cisplatin-based chemotherapy [40]. Consistent with our results, CRISPR-targeted demethylation of EphA7 works synergistically with cisplatin/paclitaxel, significantly increasing cisplatin/paclitaxel susceptibility (Fig. 5E).

Emerging data indicated that tumor DNA methylation profiling may serve as a prognostic or predictive biomarker of the response to targeted therapy and immunotherapy [41]. In addition, DNA methylation of both tumor cells and immune cells affects the TME and has been shown to play critical roles in influencing immune cell function and tumor immune evasion [42]. As a major component of the TME, immune cell infiltration and tumor purity have been proven to contribute to tumor progression and the immunotherapy response [43]. CTLs constitute a critical subset involved in adaptive immune responses [44], and immunotherapy based on CTL-mediated tumor recognition and elimination has shown remarkable anticancer efficacy [45]. Gray SM et al. reported that DNA methylation is associated with the acquisition of differential gene expression profiles unique to CTLs and that DNA methylation plays an important role in CTL differentiation and function [46]. Our results indicated that EphA7 methylation was negatively related to the infiltration score and CTL population, highlighting its prognostic role in CC (Fig. 6D).

As significant strides in immunotherapy, ICBs for recurrent or metastatic cancer have been reported [47]. However, high expression of IC genes is widely adopted as a predictor of the immunotherapeutic response rate [24]. Correlations between DNA methylation status and the expression of IC molecules and their prognostic significance have been demonstrated in multiple cancers [48, 49]. We also found that EphA7 promoter methylation was negatively correlated with the expression of IC genes (Fig. 6F) and that patients who received anti-PD-L1 treatment and presented high EphA7 expression had a better prognosis (Fig. 6G). These results indicate that patients with EphA7 demethylation are more likely to benefit from immunotherapy. These findings provide valuable information for realizing more precise and personalized immunotherapy in the future. In addition, the TMB and MSI status serve as promising pan-cancer predictive biomarkers, guiding immunotherapy in the era of precision medicine. A higher TMB leads to more neoantigens, increasing the likelihood of T-cell recognition, and correlates with better ICBs outcomes [50]. In CC, high-frequency MSI independently predicts clinical characteristics and prognosis [51]. Therefore, these insights may lead to the proposal of new targets for the development of immunosuppressants for CC treatment.

**Fig. 7:**
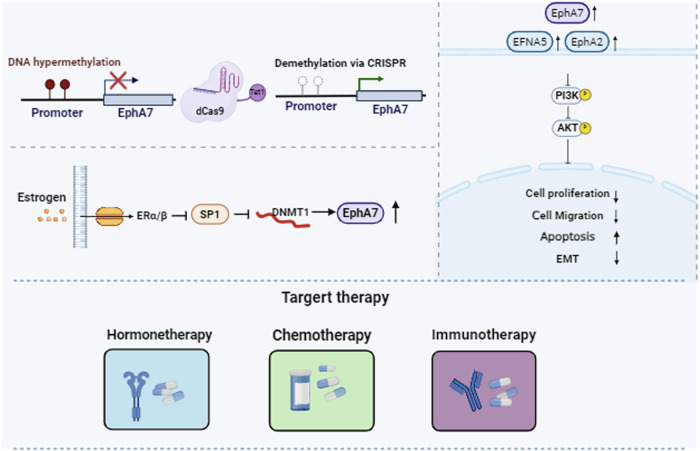
Targeted demethylation of EphA7 inhibits tumorigenesis via the SP1/DNMT1 & PI3K/AKT axis and improves the response to multiple therapies in cervical cancer. As in our previous research, EphA7 was significantly hypermethylated in CC tissues, and the CRISPR-dCas9-Tet1 system was used to reduce the promoter methylation of EphA7 by reactivating its expression, which provided strong evidence that promoter methylation of EphA7 is inversely correlated with gene expression in CC. In this study, we found that the promoter demethylation of EphA7 via the CRISPR-dCas9-Tet1 system inhibited proliferation, migration and EMT, and induced apoptosis through the PI3K/AKT signaling pathway. Moreover, we found that E2 could impair SP1, reduce the methylation of EphA7, and reverse the expression of EphA7 by reducing the expression of DNMT1 via the SP1/ER axis. Furthermore, the chemosensitivity of CaSki and SiHa cells to cisplatin and paclitaxel was effectively improved with the demethylation of EphA7, and the demethylation of EphA7 was associated with a favorable response to therapy and a favorable prognosis for CC patients. In addition, pooled analysis indicated that EphA7 hypermethylation was positively correlated with tumor purity but negatively correlated with immune infiltration, CTLs and IC activity, and the expression of EphA7 was significantly positively correlated with TMB, MSI, and SNV neoantigens, suggesting a better prognosis with EphA7 demethylation/high expression. Overall, specific demethylation of the EphA7 promoter and restoration of endogenous EphA7 expression by dCas9-Tet1 hold promising therapeutic applications and offer a favorable prognosis for CC.

In conclusion, our results comprehensively revealed that EphA7 may function as a tumor suppressor in CC by restraining tumor proliferation and invasion, promoting apoptosis, and modulating the PI3K/AKT signaling pathway in CC cells. EphA7 represents a promising therapeutic target for tumor chemo-/immunotherapy in CC. These insights (Fig. 7) provide a strong foundation for identifying novel therapeutic targets for CC management, although future translational research should consider delivery systems.

## Supplementary information

Supplemental file

Supplementary Table

Original data

## Data Availability

All the data generated or analyzed during this study can be found in this published article and its supplementary information files. Only publicly available data were used for this study. The sources of these data and how we handled them are described in the Materials and Methods section. Additional data that support the findings of our study can be obtained from the corresponding author upon request.
