# Supplementary material for: Targeted demethylation of the EphA7 promoter inhibits tumorigenesis via the SP1/DNMT1 and PI3K/AKT axes and improves the response to multiple therapies in cervical cancer
Source: Cell Death Dis. 2025 Apr 21;16(1):324. doi: 10.1038/s41419-025-07512-4 (PMC12012199; doi:10.1038/s41419-025-07512-4)
Supplement: Supplementary file 1 — Supplemental file [file 41419_2025_7512_MOESM1_ESM.docx]

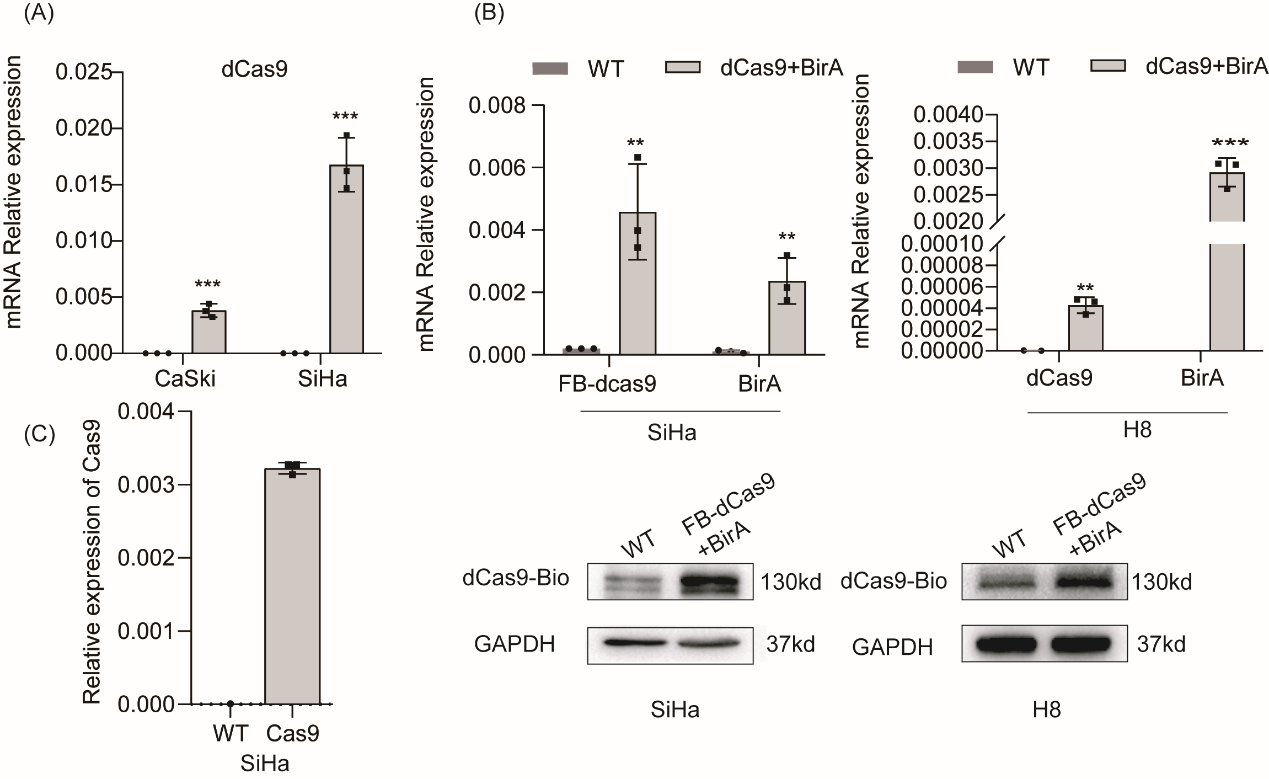


**Figure S1. Results of the identification of stable cell lines in this study. (**A) qRT‒PCR was performed to verify that the dCas9 mRNA was expressed in CaSki/SiHa-dCas9-Tet1 cells but not in wild-type (WT) cells. (B) The mRNA expression of dCas9 and BirA was detected in SiHa/H8-FB-dCas9+BirA cells via qRT‒PCR (top), and the expression of biotin-modified dCas9 protein in FB-dCas9+BirA cells was upregulated compared with that in WT SiHa/H8 cells via western blotting (bottom). (C) Compared with that in WT cells, the Cas9 mRNA expression in SiHa-Cas9 cells was obviously increased.


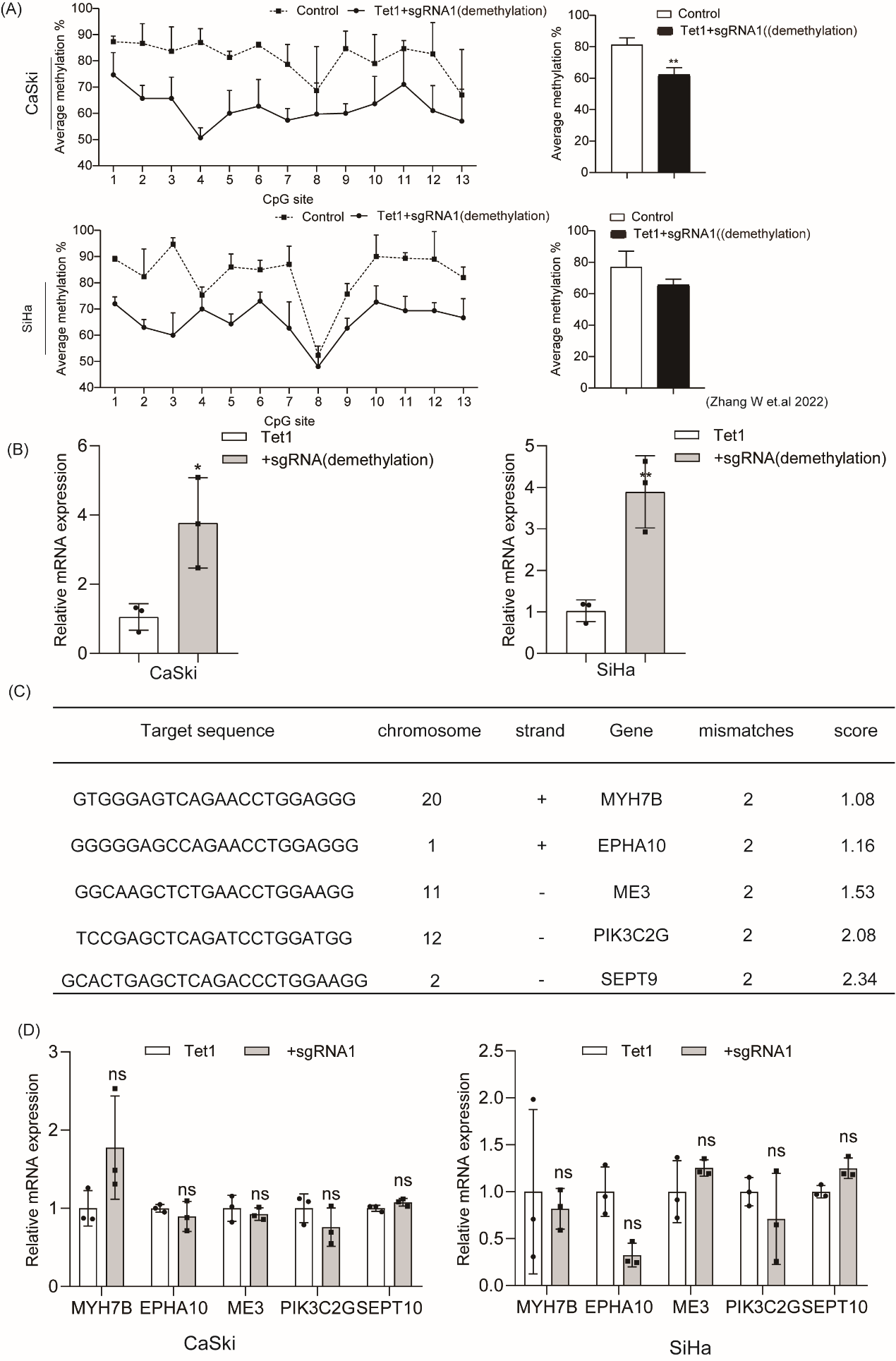
**Figure S2. Reactivation of EphA7 expression with demethylation in cervical cancer cells via the dCas9-Tet1 system.** (A) Pyrosequencing revealed that EphA7 promoter methylation was lower in both CaSki and SiHa cells in the dCas9-Tet1 group than in those in the control group. Reprinted with permission from ref.6. Copyright (2022) BMC Cancer under Creative Commons Attribution 4.0 International License (http://creativecommons.org/licenses/by/4.0/). (B) qRT‒PCR results revealed that the expression of EphA7 was restored in CaSki and SiHa cells (*p*<0.05). (C) To evaluate the off-target effects of the dCas9-Tet1 demethylation system, the top 5 potential sites predicted for act-sgRNA1 were selected. (D) The top 5 potential off-target loci predicted by the COSMID web tool (https://crispr.bme.gatech.edu/) were selected. The results of qRT‒PCR revealed that off-target mRNA expression was not significantly different between the experimental and control groups of CaSki and SiHa cells (*n=*3). * *p*<0.05, ** *p*<0.01, ns, not significant.


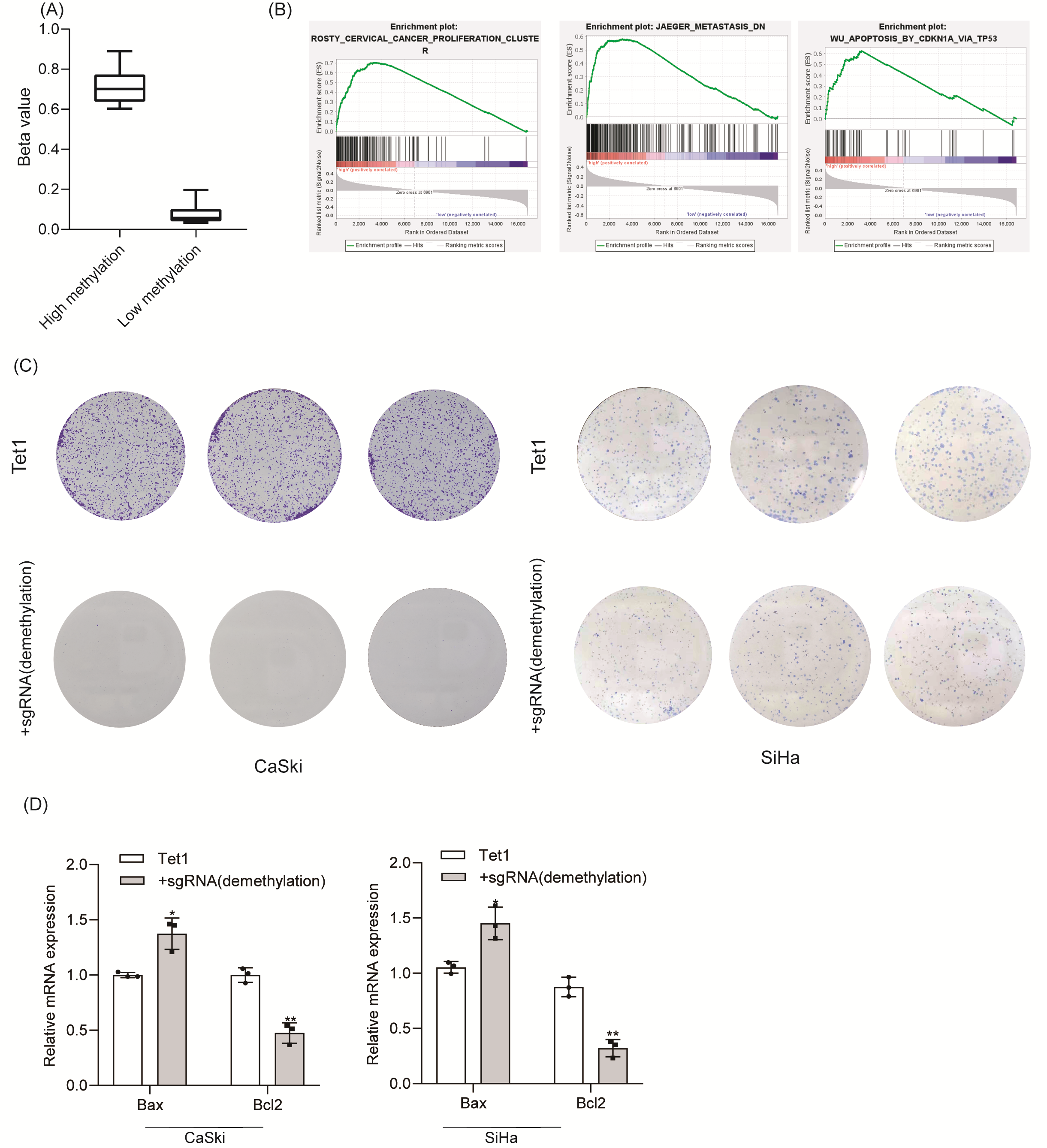


**Figure S3. The demethylation of EphA7 via the dCas9-Tet1 system inhibited CC progression and induced apoptosis.** (A) Two groups were generated according to the promoter methylation of EphA7 based on the TCGA datasets. (B) The potential biological functions of EphA7 methylation were investigated via gene set enrichment analysis (GSEA) software (https://www.broadinstitute.org/gsea/). (C) Colony formation assays revealed that EphA7 demethylation decreased the number and size of colonies in CaSki and SiHa cells compared with those in the control group. (D) Compared with that in the control group, the mRNA expression of Bax was significantly increased (*p*<0.05), whereas that of Bcl-2 was downregulated (*p*<0.05), as determined via qRT‒PCR. * *p*<0.05, ** *p*<0.01, *** *p*<0.001.


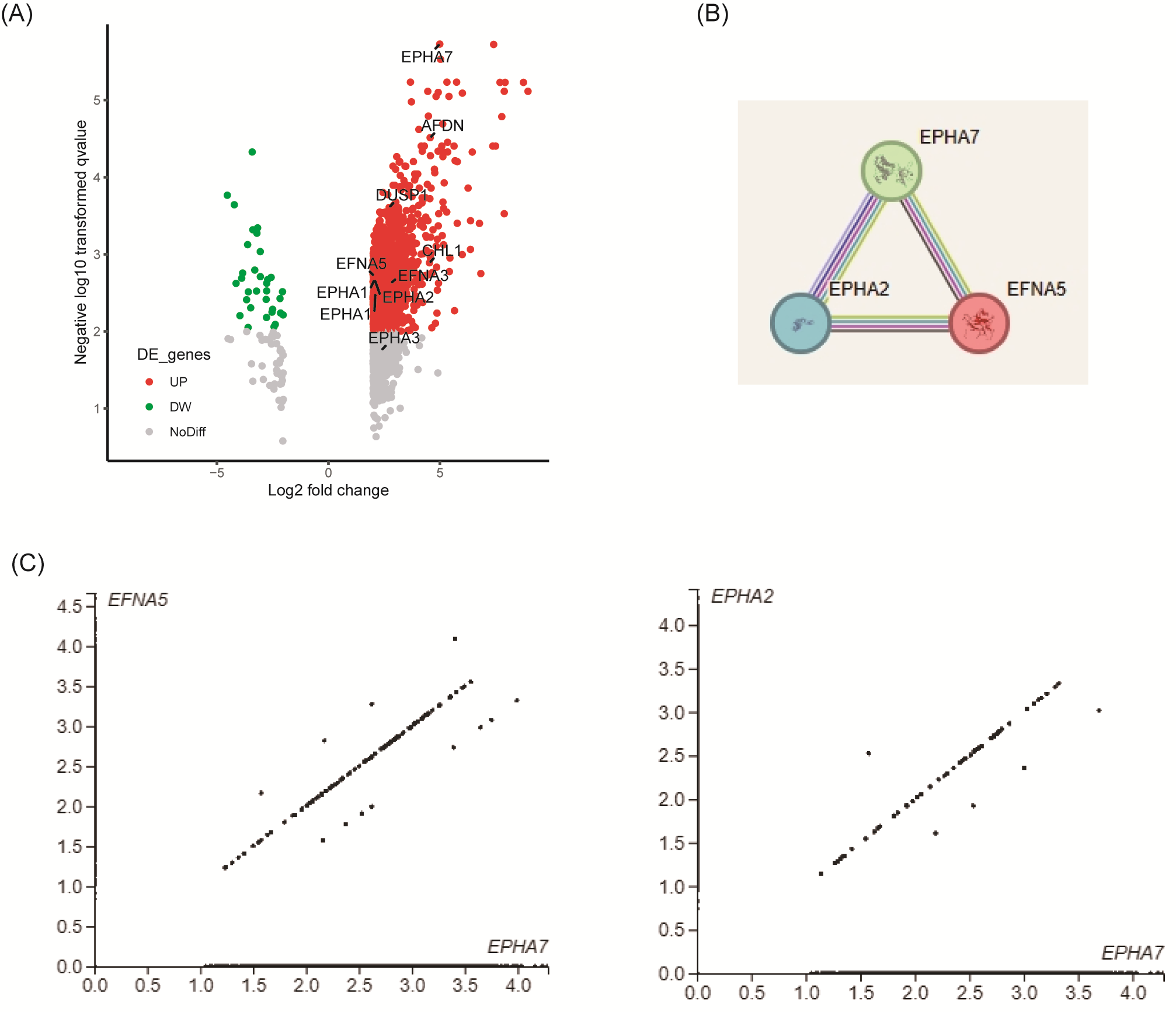


**Figure S4. Correlation analysis of the correlations between the target gene EphA7 and its related genes.** (A) DEGs for EphA7 from the comparison of the cervical normal samples with high EphA7 expression (*n=*5) and the cervical cancer samples with low EphA7 expression. (B) The protein database (STRING) revealed that the EphA7 protein could directly bind to EFNA5 and EphA2. (C) scRNA-seq revealed that EphA7 expression was positively associated with EFNA5 and EphA7 expression in human cells via CellXGene tools.


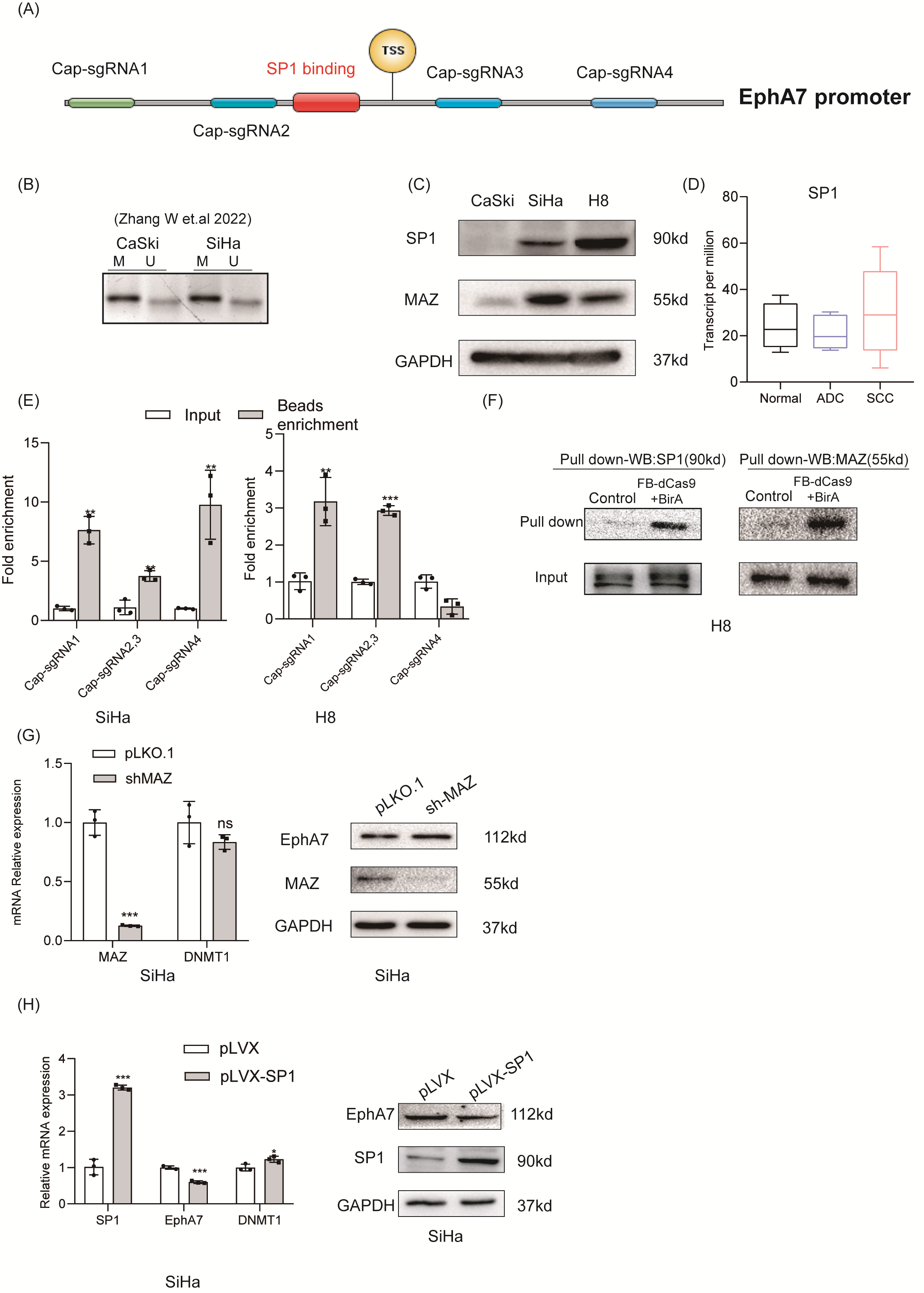


**Figure S5. The** **on-target enrichment of capture sgRNA and the overexpression of SP1 decreased the expression of EphA7.** **(A)** The site of the sgRNAs used for capture in the promoter of EphA7. **(B)** MSP was performed to test the promoter methylation of EphA7 in CaSki and SiHa cells. Reprinted with permission from ref.6. Copyright (2022) BMC Cancer under Creative Commons Attribution 4.0 International License (http://creativecommons.org/licenses/by/4.0/). **(C)** Protein expression of total SP1 and MAZ in CaSki, SiHa and H8 cells**. (D)** Analysis revealed that SP1 has a lower level in the ADC than in the SCC in CESCs via UACLAN**. (E)** Cap-sgRNA1-4 effectively enriched the fragments of the EphA7 promoter in both SiHa and H8 cells (*p*<0.05). **(F)** The in situ pull-down results verified that SP1 and MAZ located in the promoter region of EphA7 in H8 cells. **(G)** Knockout of MAZ did not affect the expression of DNMT1 or EphA7. **(H)** Compared with that in the control group (pLVX), the mRNA expression of SP1 and DNMT1 was upregulated (*p*<0.05), whereas the expression of EphA7 was reduced (*p*<0.05) in SiHa-SP1-overexpressing cells (pLVX-SP1), and the protein expression of EphA7 was downregulated with the overexpression of SP1 compared with that in the control group. * *p*<0.05, ** *p*<0.01, *** *p*<0.001.


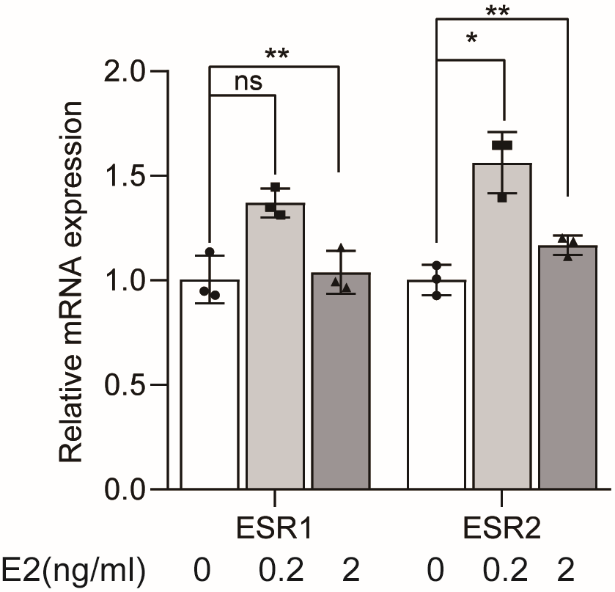


**Figure S6.** The mRNA expression of ESR1 and ESR2 generally increased after SiHa cells were treated with E2 (0.2 ng/ml and 2 ng/ml) (*p*<0.05). * *p*<0.05, ** *p*<0.01, and ns, not significant.


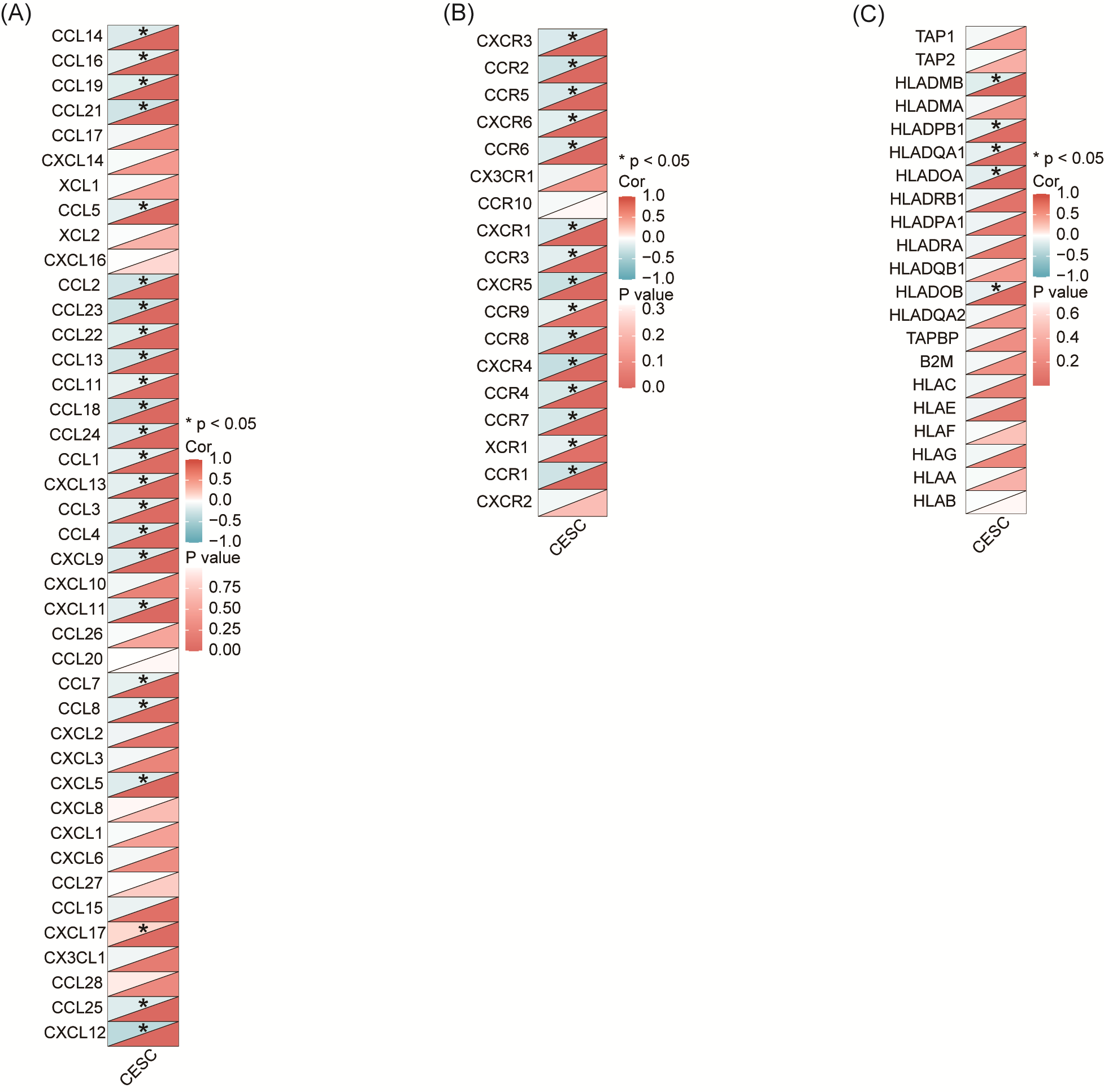


**Figure S7. Correlation of EphA7 methylation with immune-related genes.** (A) Chemokines. (B) Chemokine receptors. (C) MHC genes. The color scales indicate Spearman’s correlation coefficient and the *p* value. The upper triangle represents the *p* value, and **p* < 0.05. The lower triangle in the heatmap represents the correlation coefficient. Red indicates a positive correlation, and blue indicates a negative correlation.
