# Supplementary material for: Targeted demethylation of the EphA7 promoter inhibits tumorigenesis via the SP1/DNMT1 and PI3K/AKT axes and improves the response to multiple therapies in cervical cancer
Source: Cell Death Dis. 2025 Apr 21;16(1):324. doi: 10.1038/s41419-025-07512-4 (PMC12012199; doi:10.1038/s41419-025-07512-4)

Fig.S1B

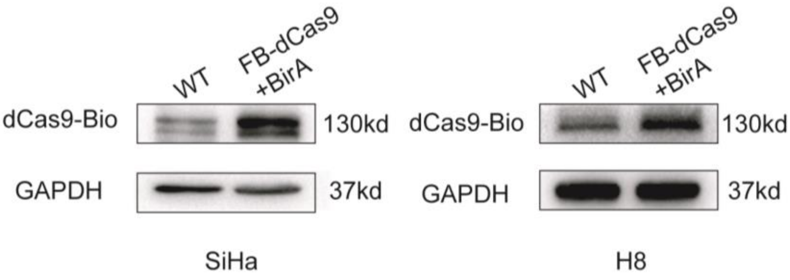

dCas9-bio (130kd)

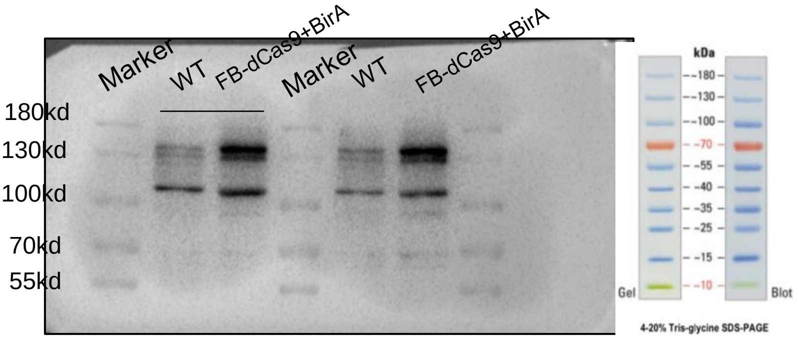

SiHa

dCas9-bio (130kd)

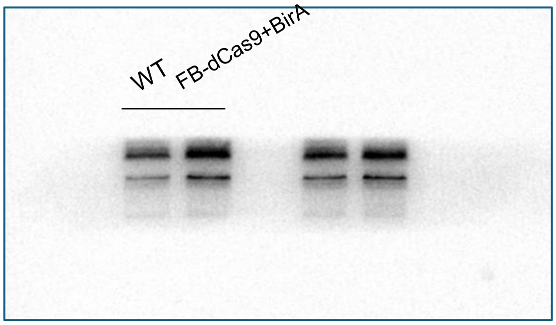

H8

GAPDH (37kd)

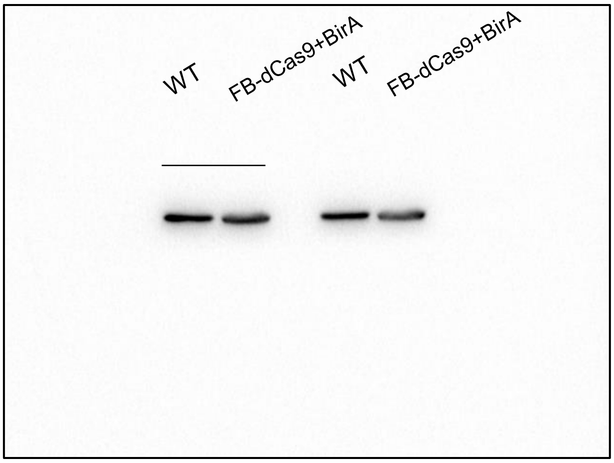

SiHa

GAPDH (37kd)

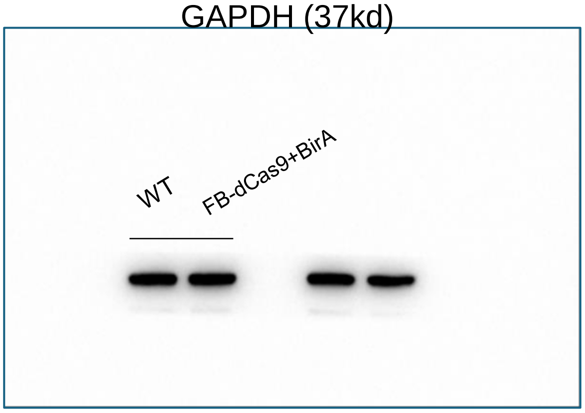

H8

Fig.1A

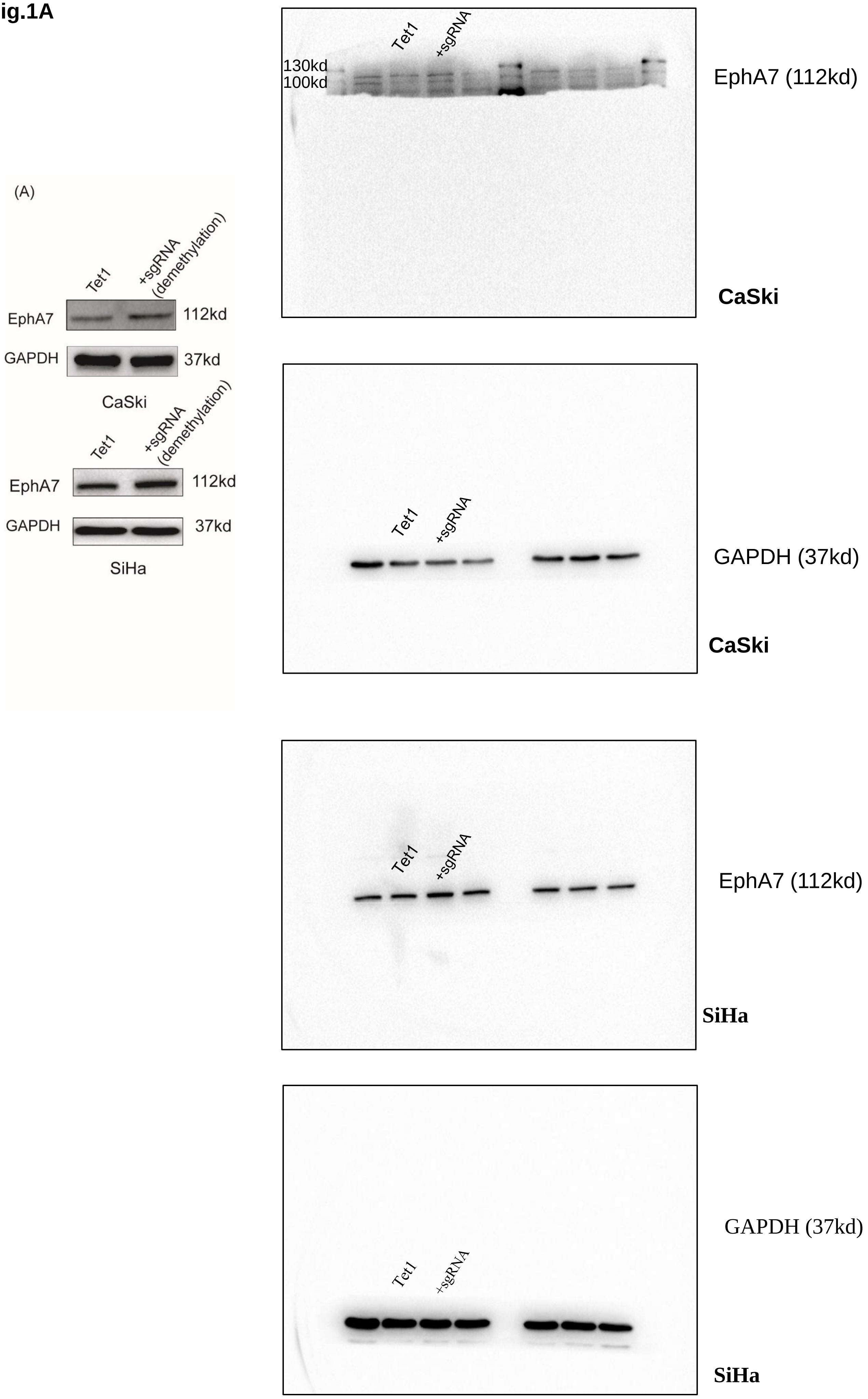

(A)
Tet1
+sgRNA (demethylation)
EphA7
112kd
GAPDH
37kd
CaSki
Tet1
+sgRNA (demethylation)
EphA7
112kd
GAPDH
37kd
SiHa
Tet1
+sgRNA
130kd
100kd
EphA7 (112kd)
CaSki
Tet1
+sgRNA
GAPDH (37kd)
CaSki
Tet1
+sgRNA
EphA7 (112kd)
SiHa
Tet1
+sgRNA
GAPDH (37kd)
SiHa

Fig.1C

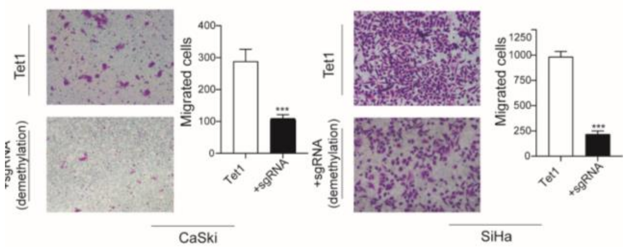

Tet1

+sgRNA

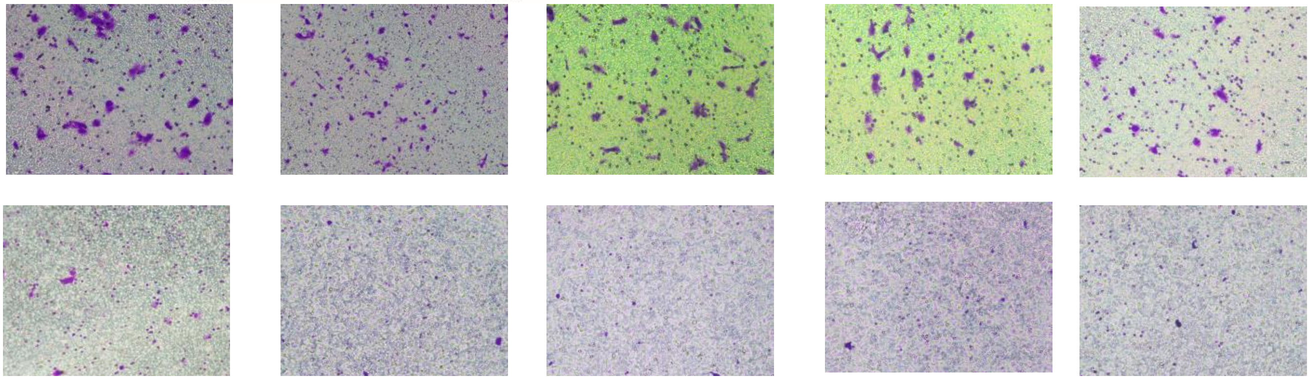

CaSki

Tet1

+sgRNA

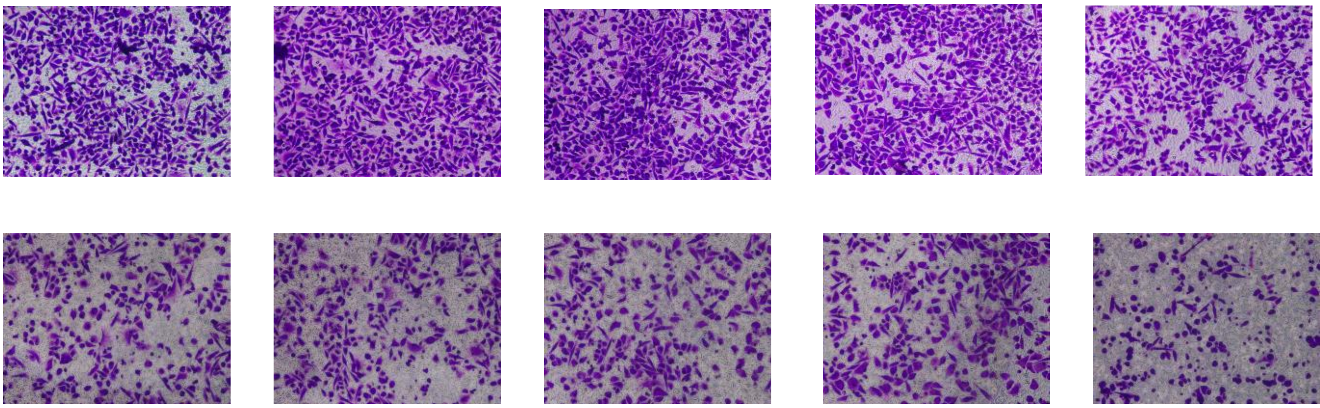

SiHa

Fig.1E

Figure 1

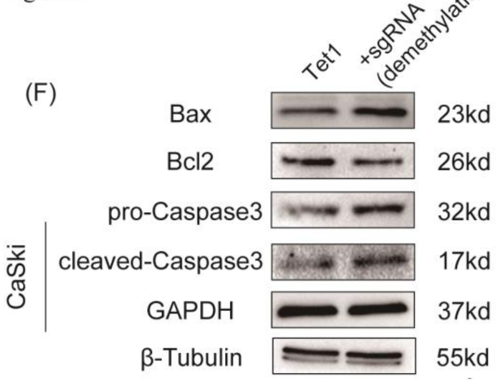

CaSki

Bax (23kd)

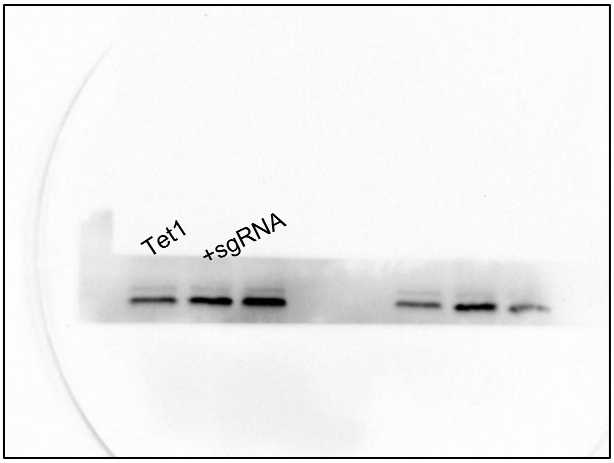

Bcl2 (26kd)

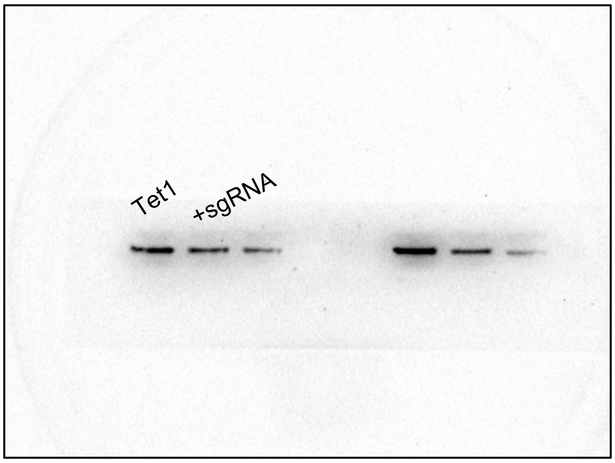

Pro-Caspase3 (32kd)

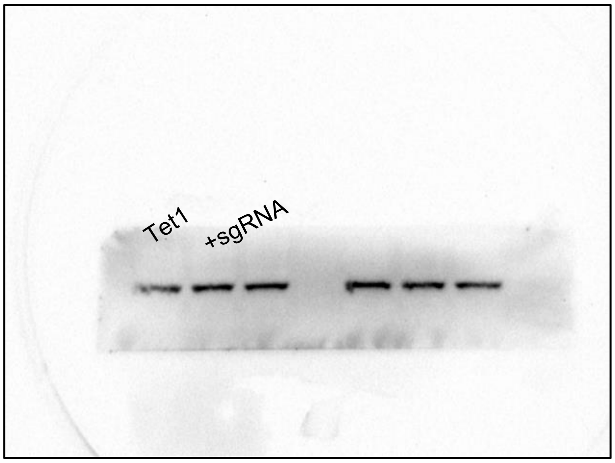

GAPDH (37kd)

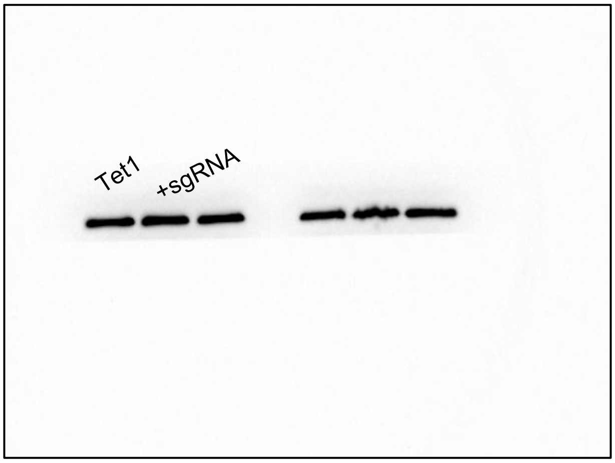

cleaved-Caspase3 (17kd)

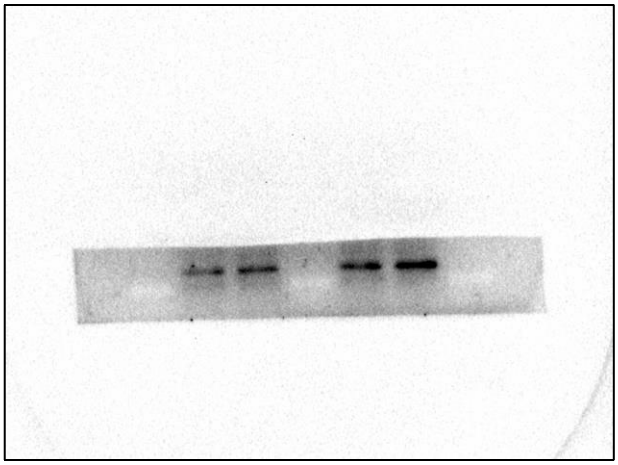

β-Tubulin (55kd)

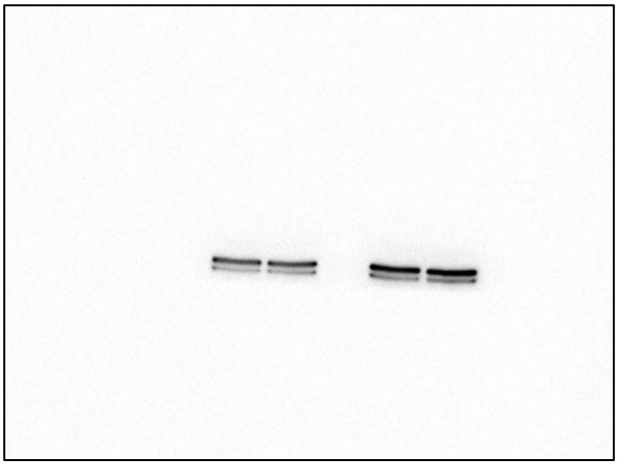

Fig.1E

SiHa

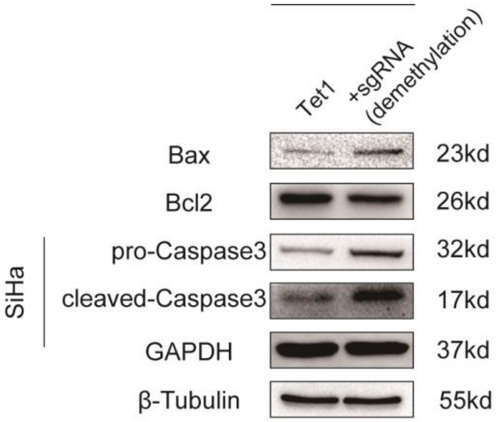

Bax (23kd)

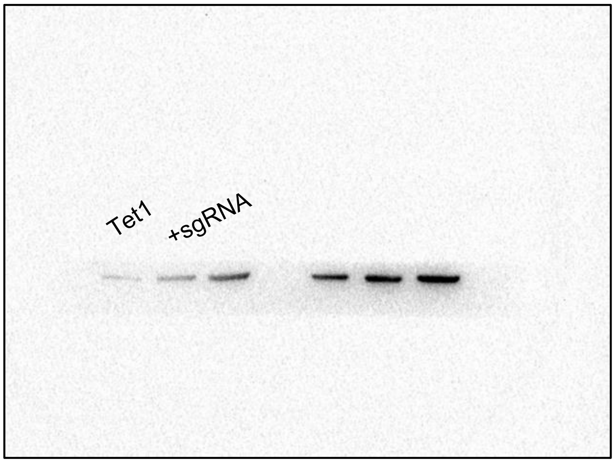

Bcl2 (26kd)

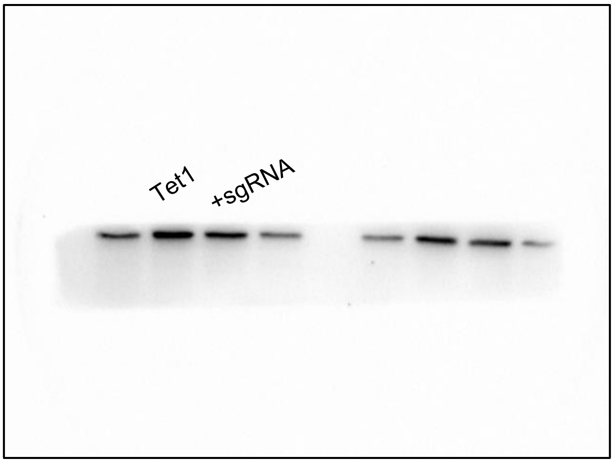

Pro-Caspase3 (32kd)

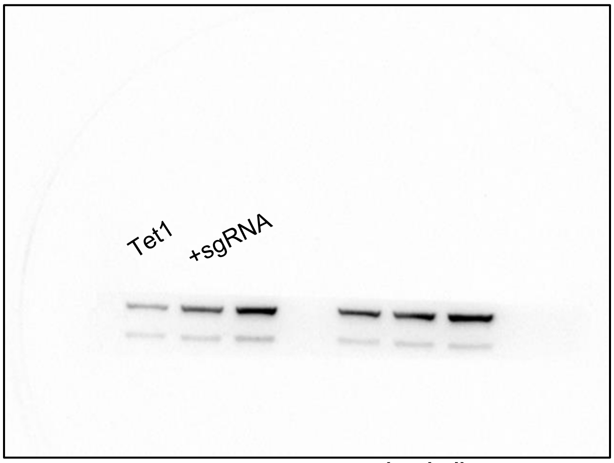

GAPDH (37kd)

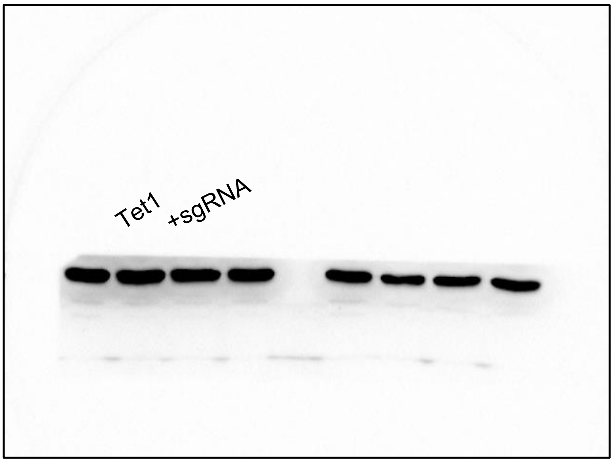

GAPDH (37kd)

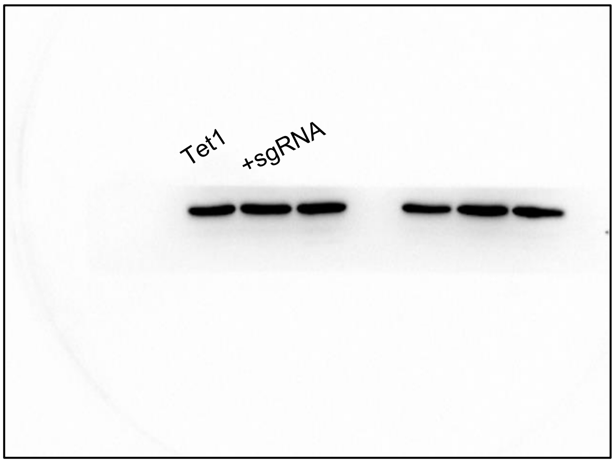

cleaved-Caspase3 (17kd)

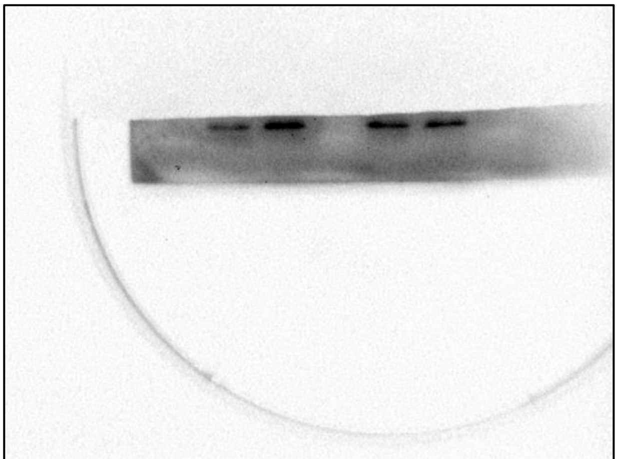

β-Tubulin (55kd)

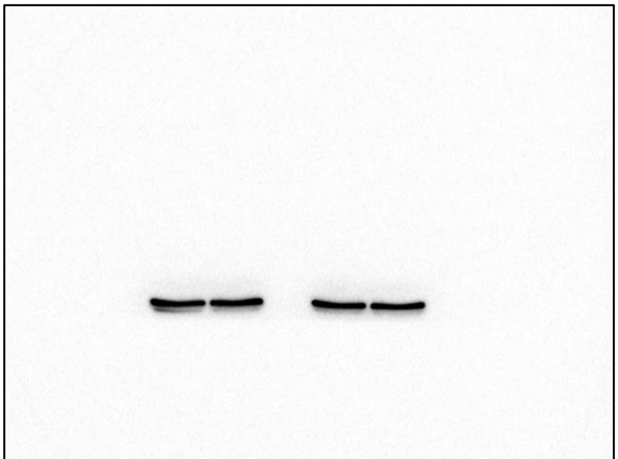

Fig.2C

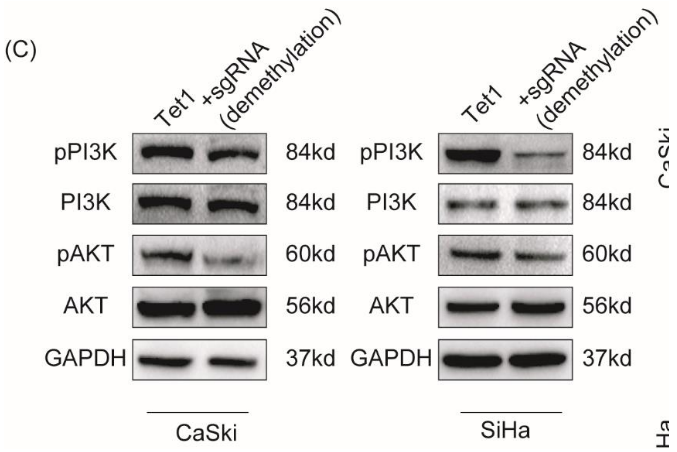

CaSki

pAKT (60kd)

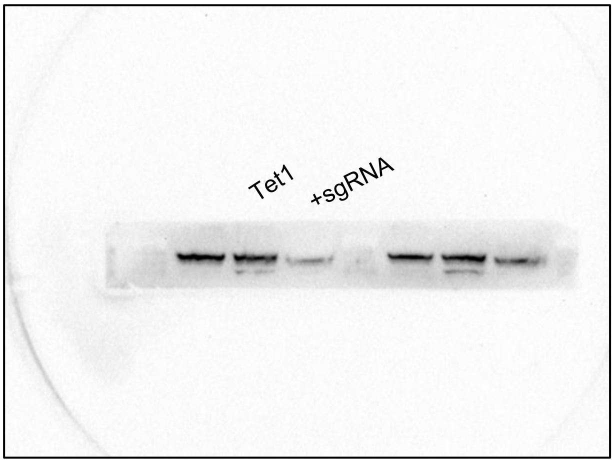

pPI3K (84kd)

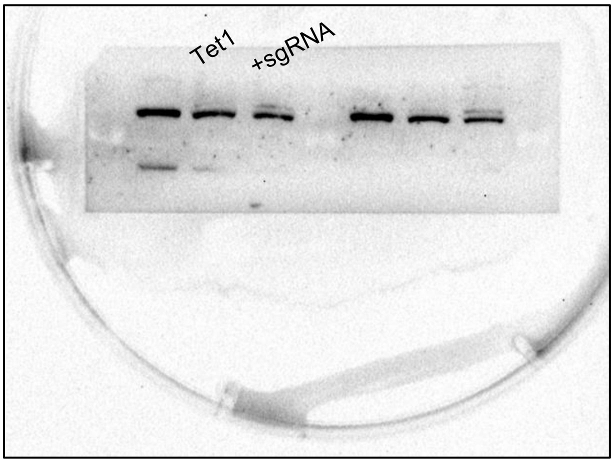

AKT (56kd)

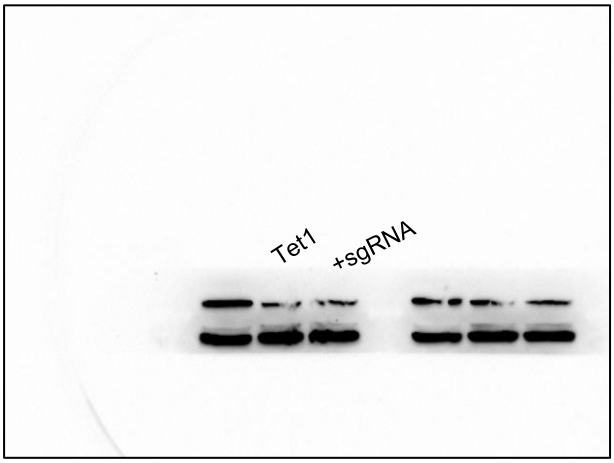

PI3K (84kd)

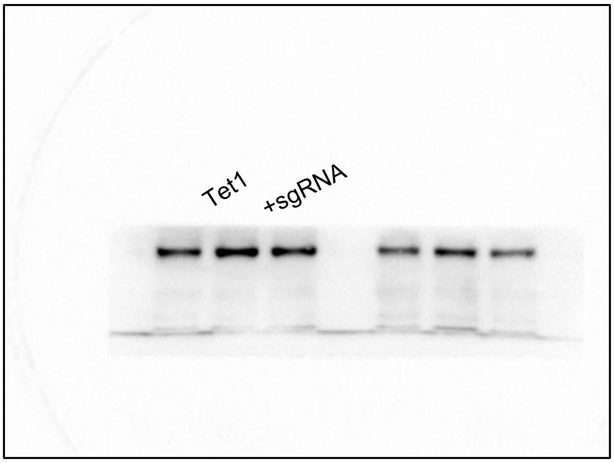

GAPDH (37kd)

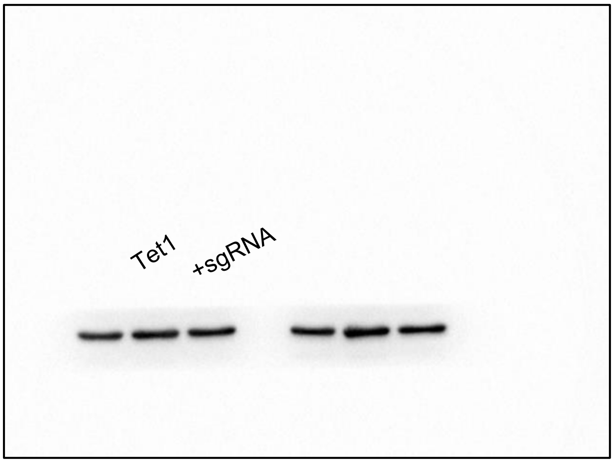

Fig.2C

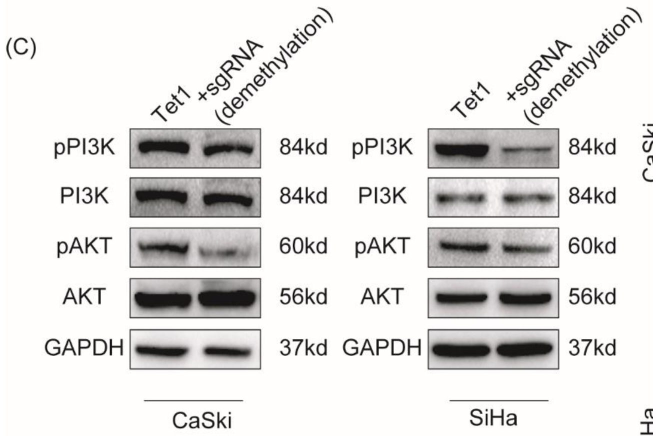

SiHa

pAKT (60kd)

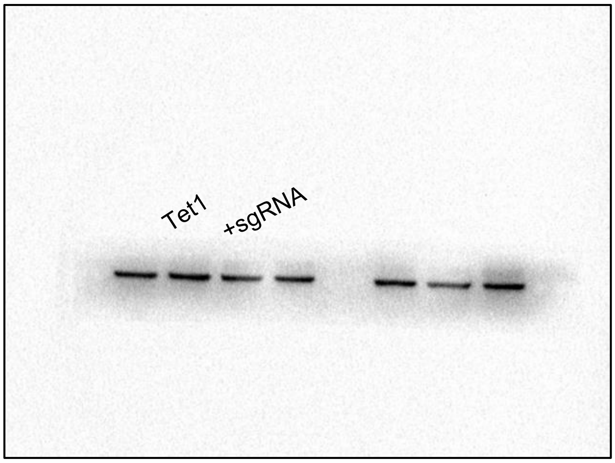

pPI3K (84kd)

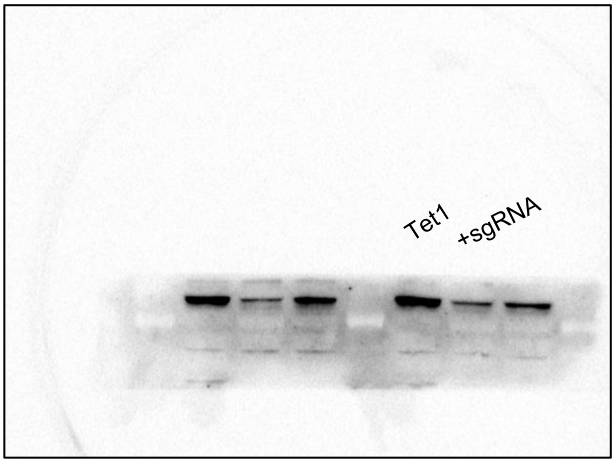

AKT (56kd)

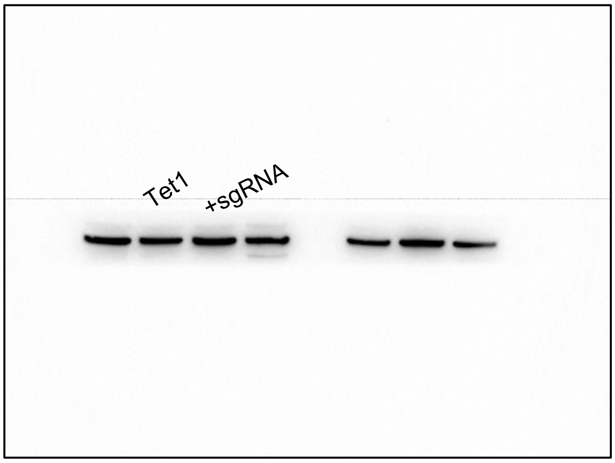

PI3K (84kd)

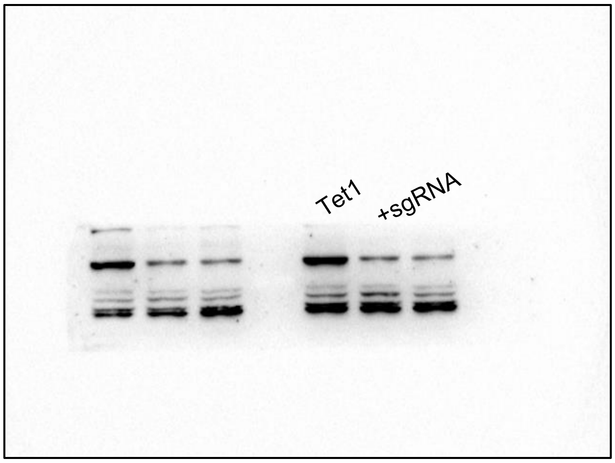

GAPDH (37kd)

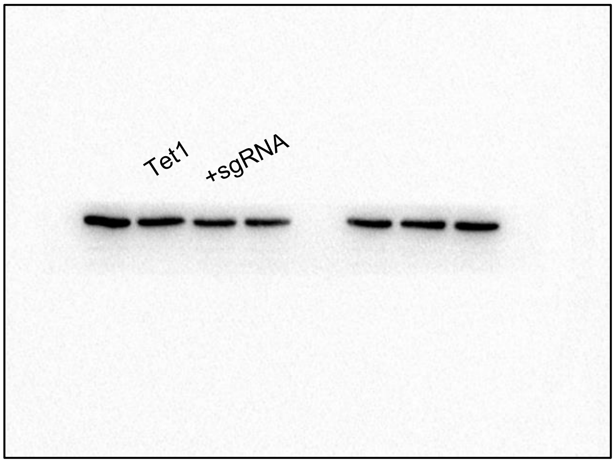

GAPDH (37kd)

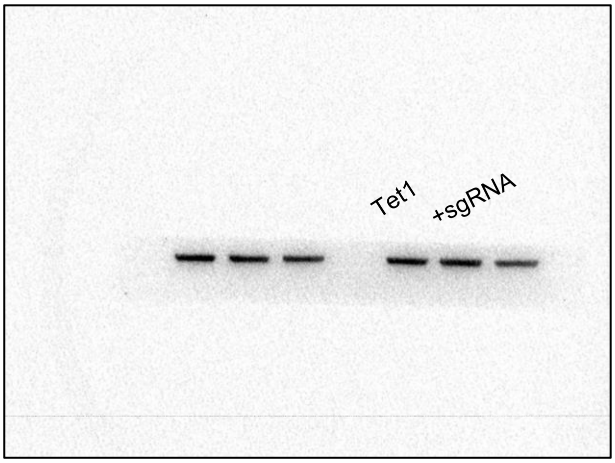

Fig.3B and 3C

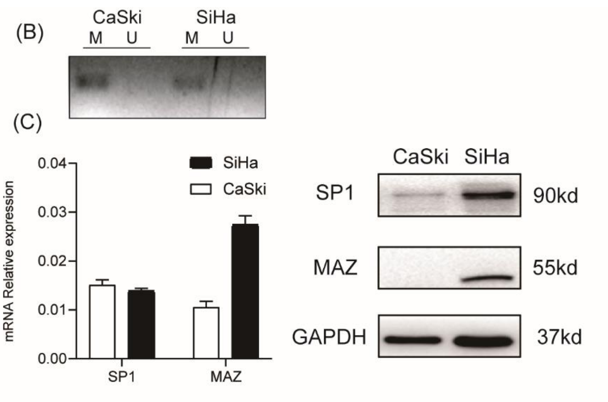

SP1 (90kd)

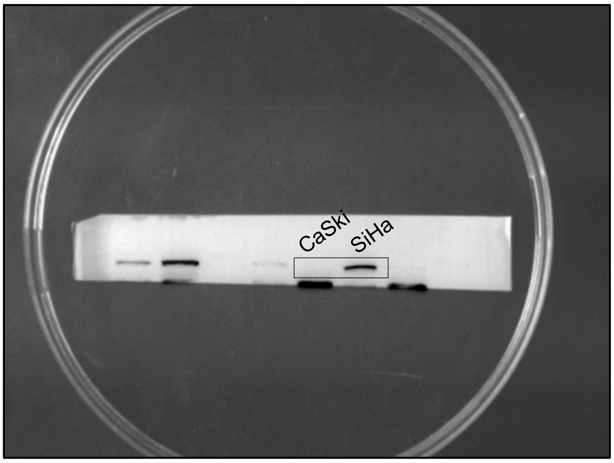

EphA7-MSP

M:229bp ;U:229bp

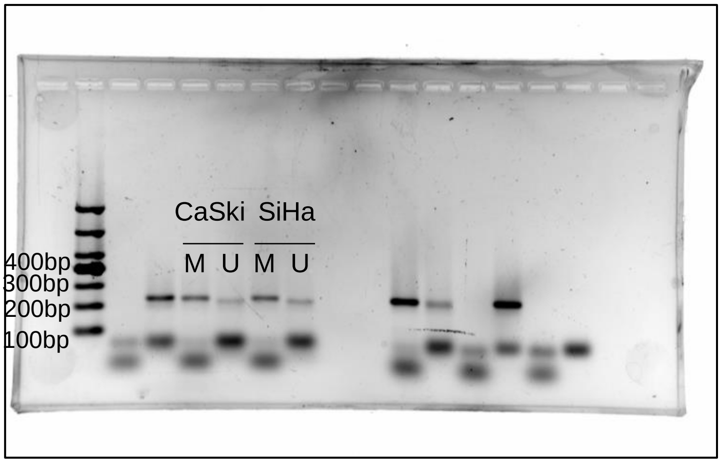

MAZ1 (55kd)

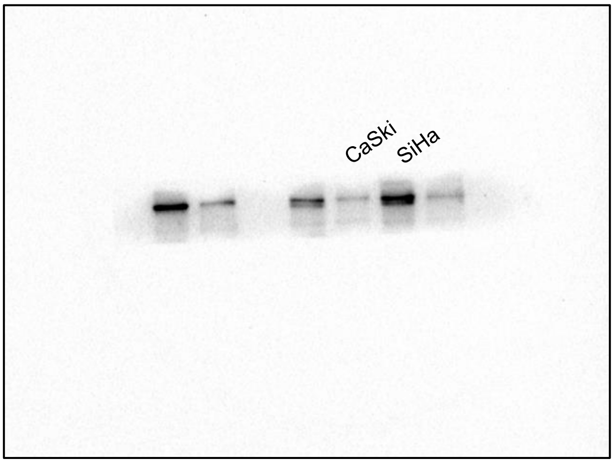

GAPDH (37kd)

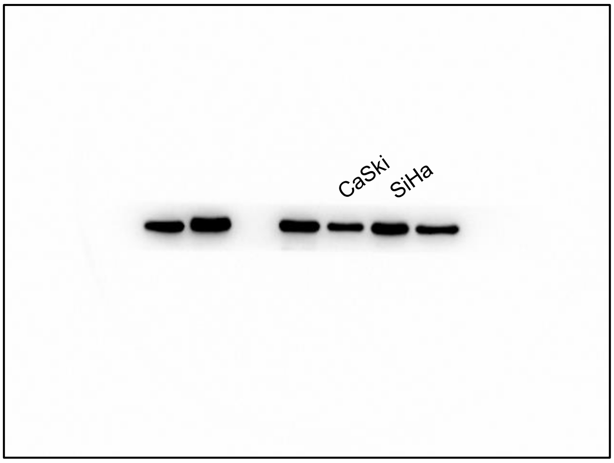

Fig.3D

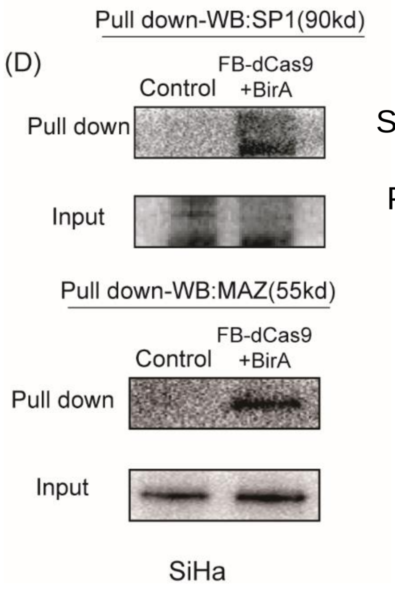

SP1 (90kd)

Pull down

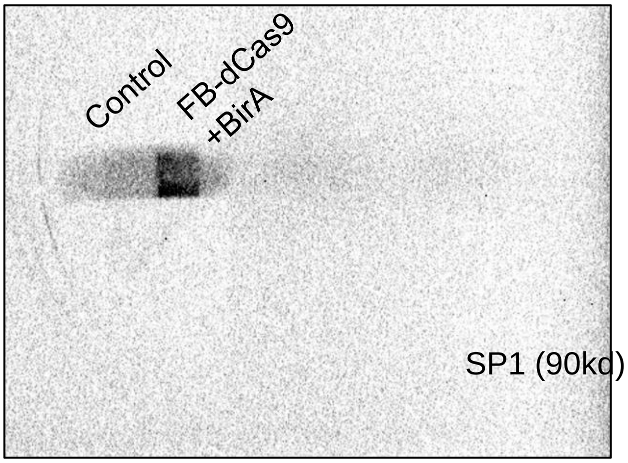

SP1 (90kd)

Input

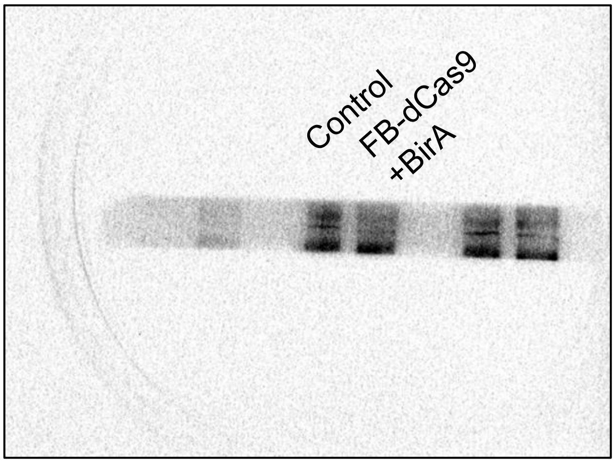

MAZ1 (55kd)

Pull down

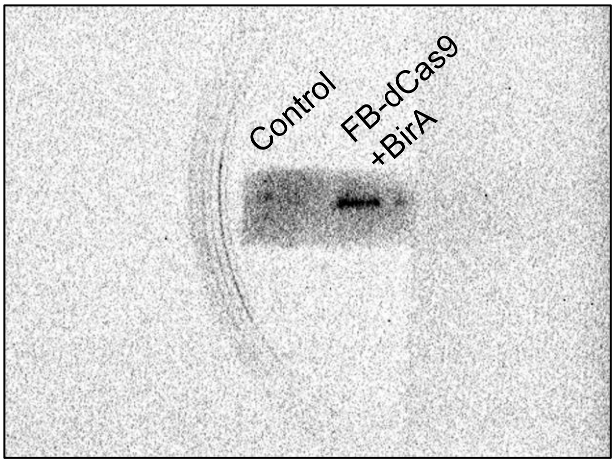

MAZ1 (55kd)

Input

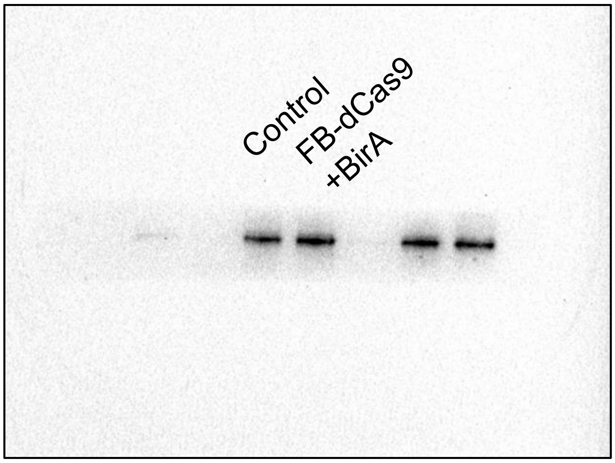

Fig.S5E

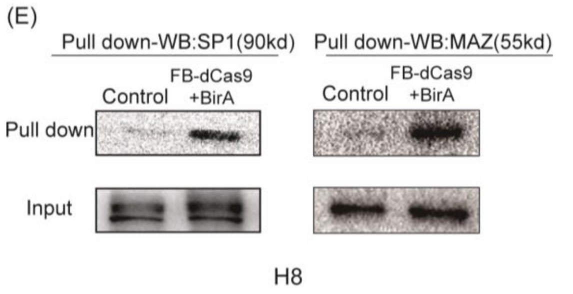
(E)
Pull down-WB:SP1(90kd)
Pull down-WB:MAZ(55kd)
Control
FB-dCas9
+BirA
Pull down
Input
H8

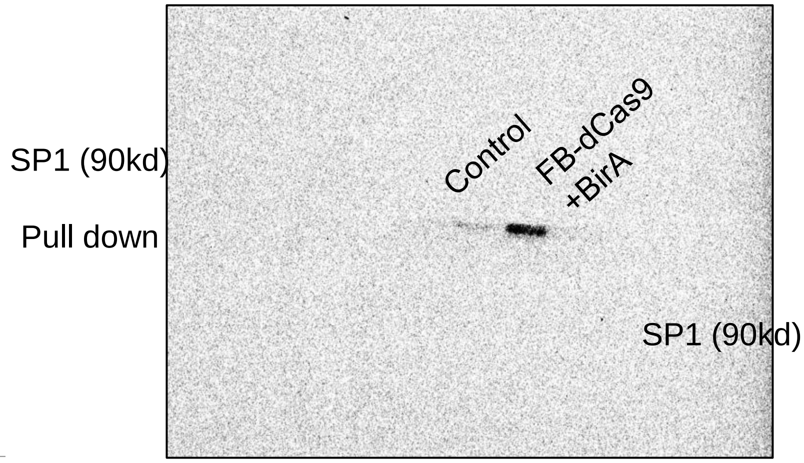
SP1 (90kd)
Pull down
Control
FB-dCas9
+BirA
SP1 (90kd)

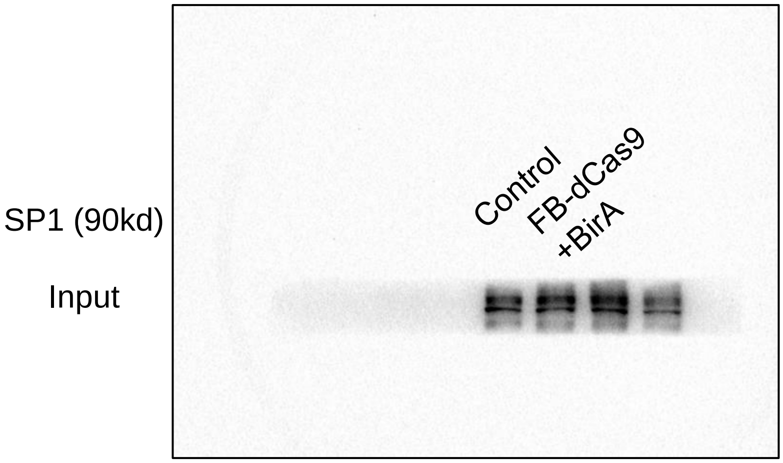
SP1 (90kd)
Input
Control
FB-dCas9
+BirA

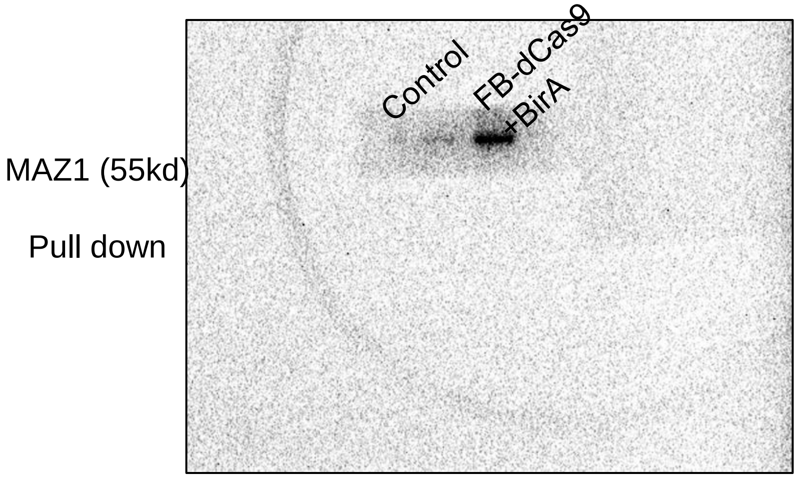
MAZ1 (55kd)
Pull down
Control
FB-dCas9
+BirA

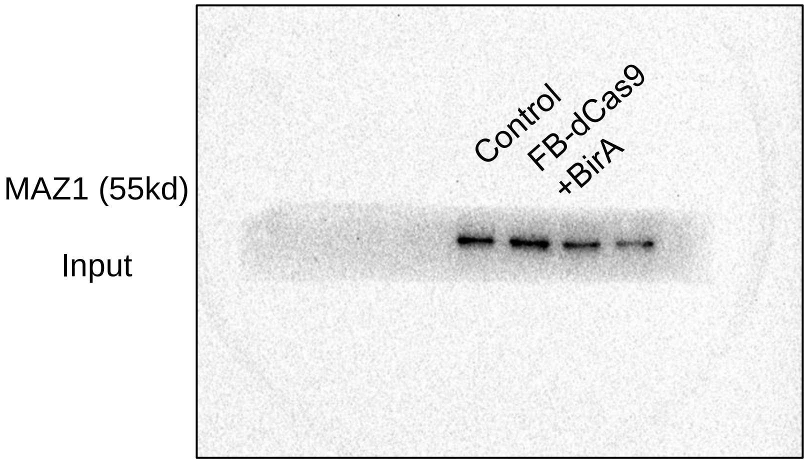
MAZ1 (55kd)
Input
Control
FB-dCas9
+BirA

Fig.3F

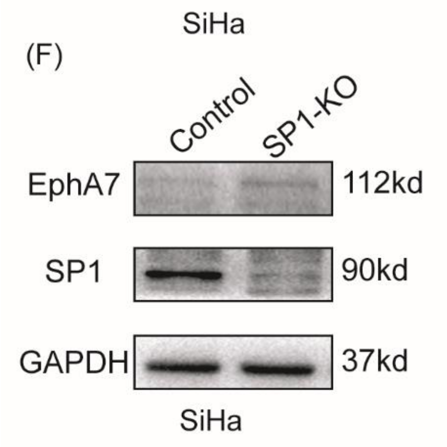

EphA7 (112kd)

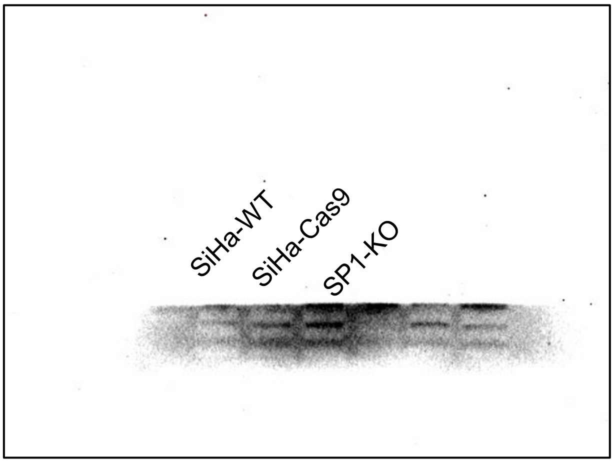

SP1(90kd)

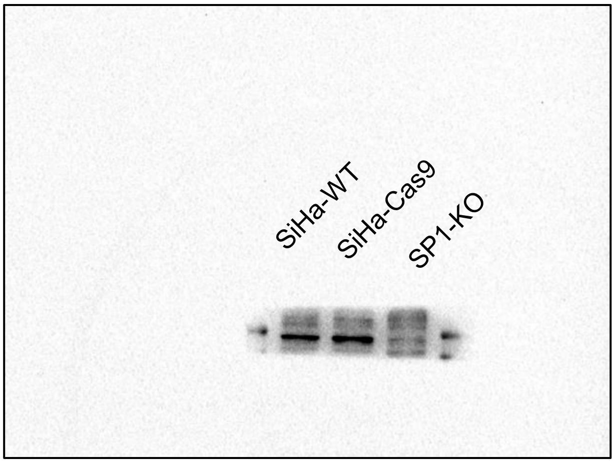

GAPDH (90kd)

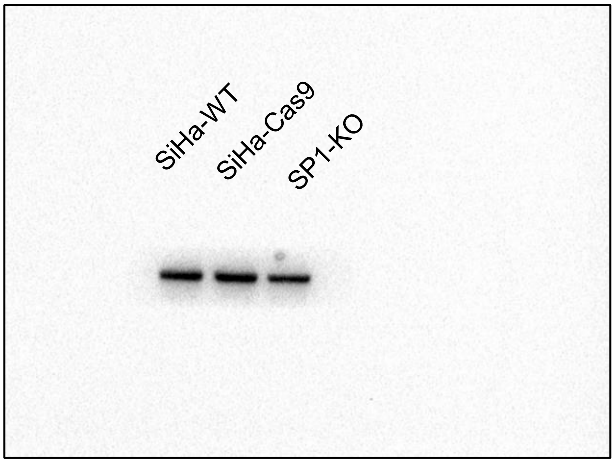

Fig. 3H

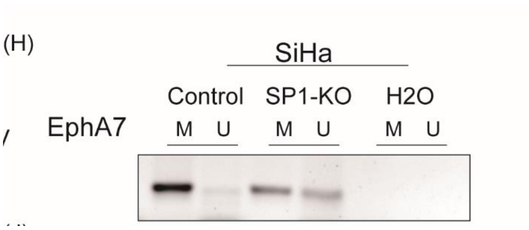

EphA7-MSP

M:229bp ;U:229bp

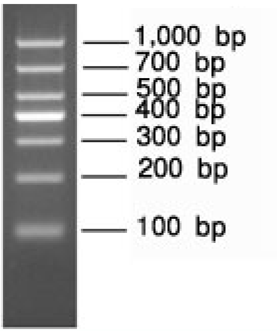

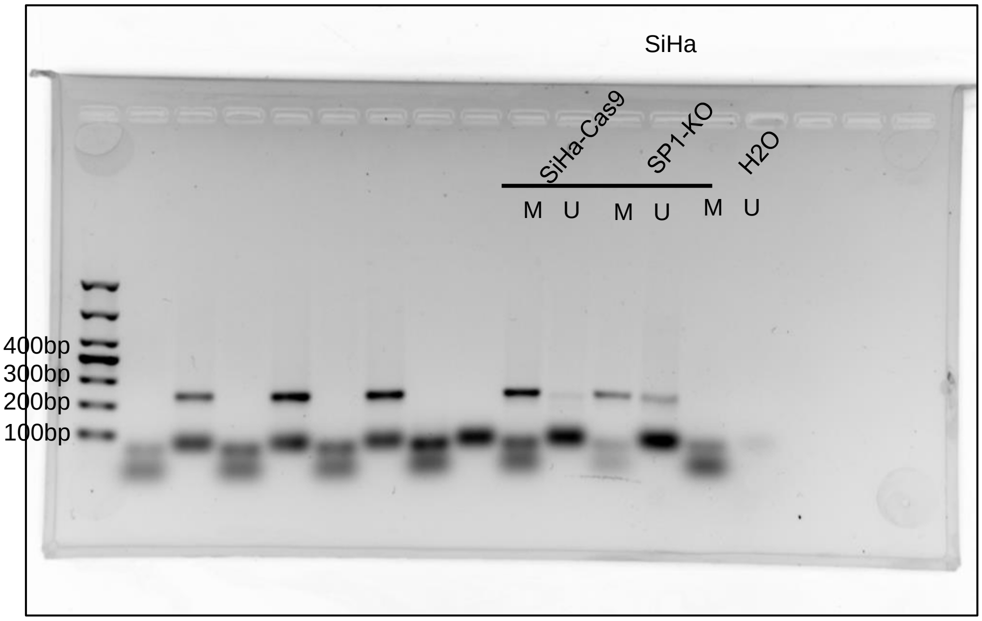

Fig. 3J

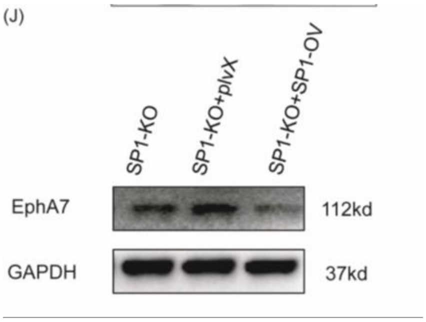

EphA7-112Kd

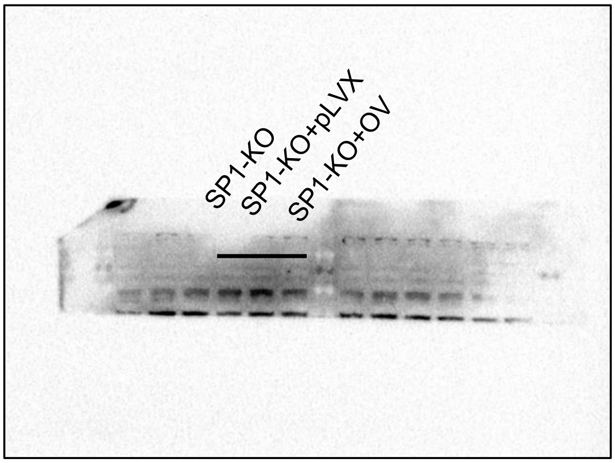

GAPDH-37Kd

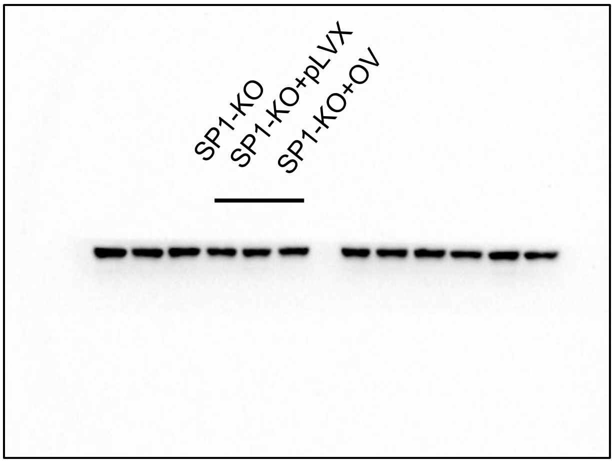

Fig.S5B

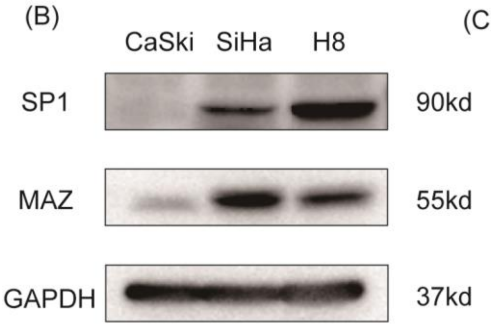

SP1 (90kd)

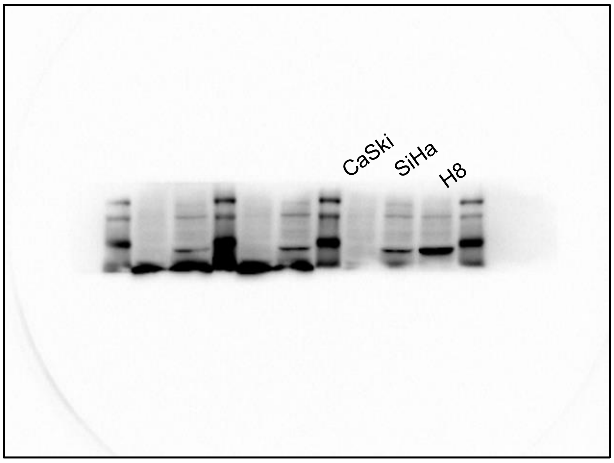

MAZ1 (55kd)

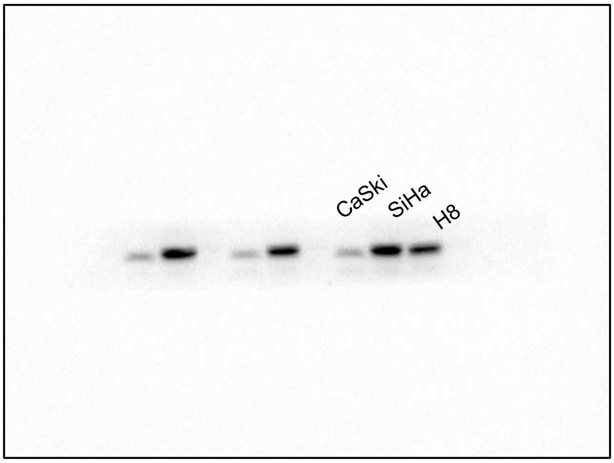

GAPDH (37kd)

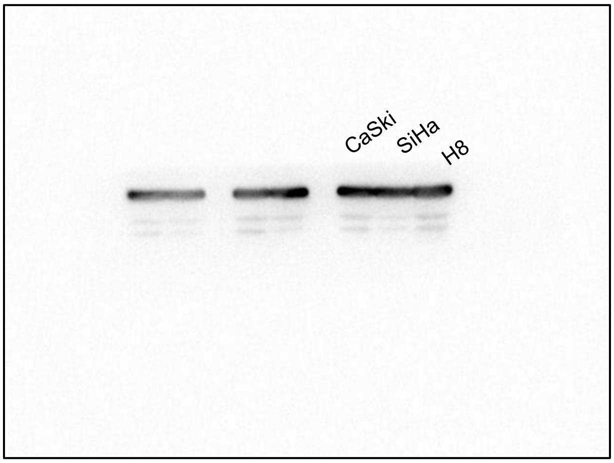

Fig.S5F

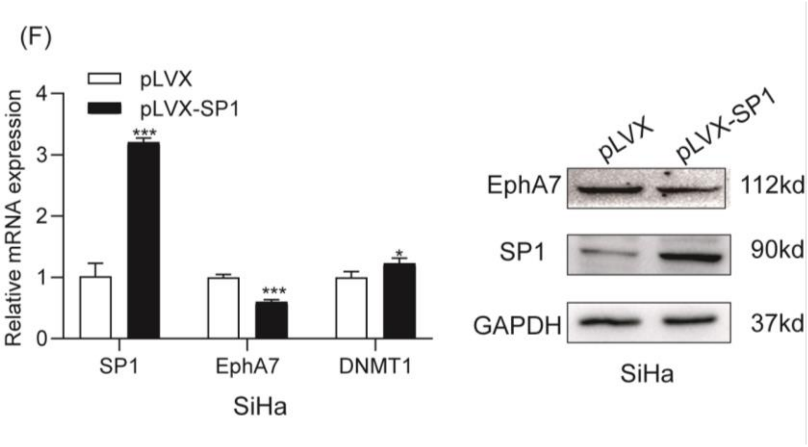

EphA7-112Kd

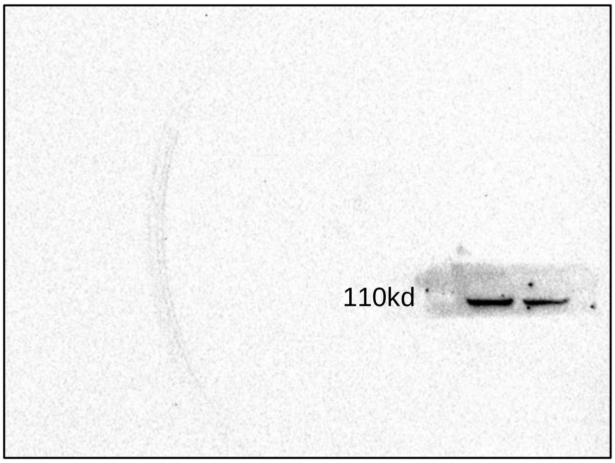

SP1-90Kd

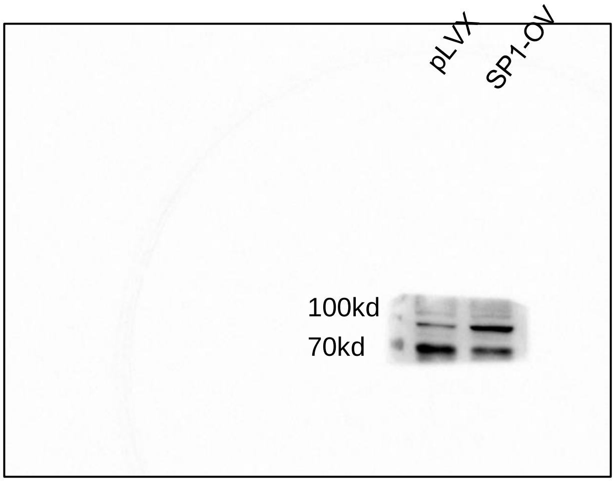

GAPDH-37Kd

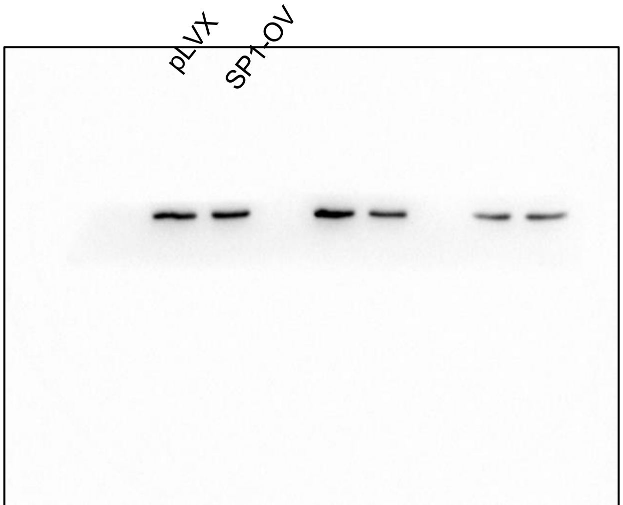

Fig.S5G

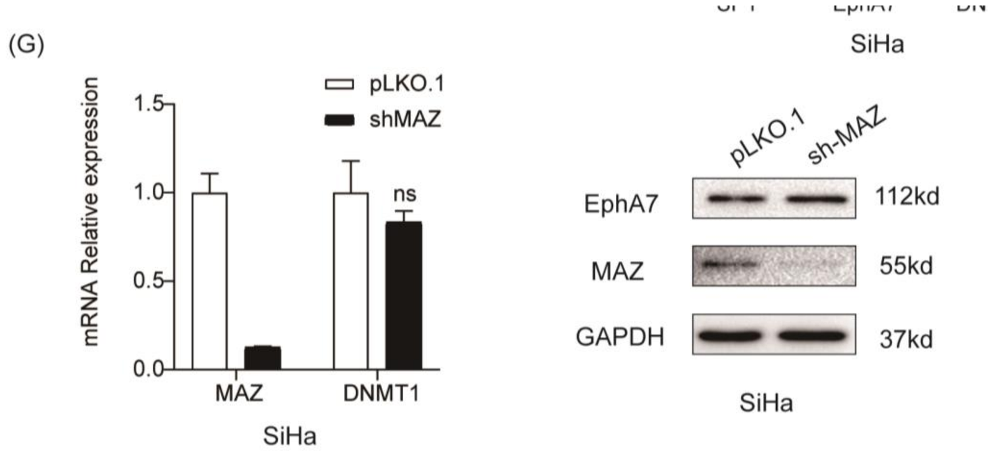

EphA7-112Kd

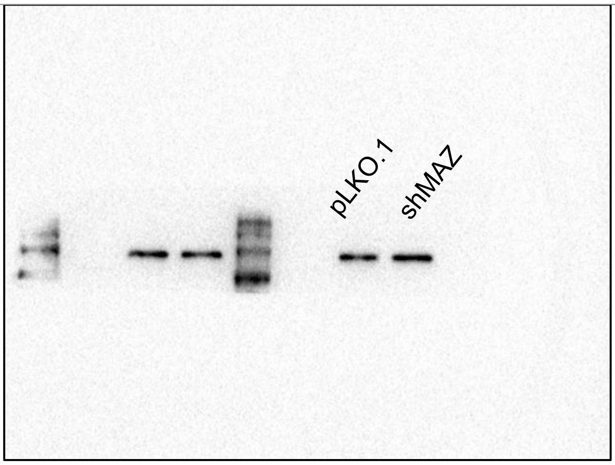

SP1-90Kd

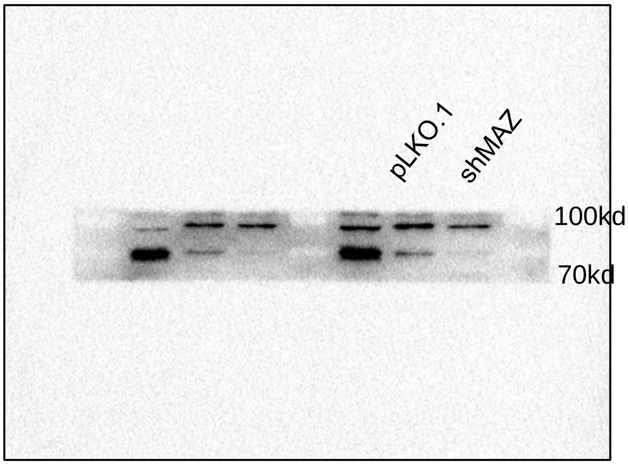

GAPDH-37Kd

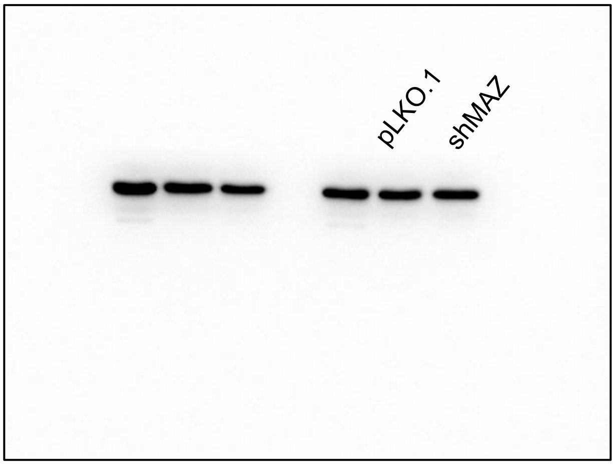

Fig.4C

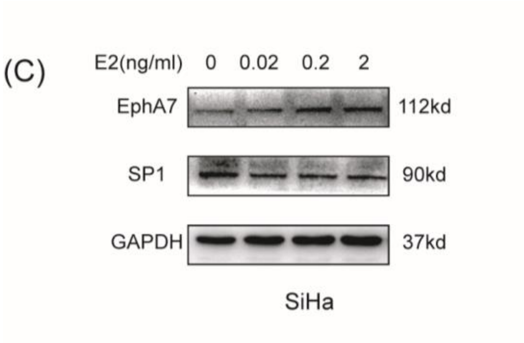

E2(ng/ml) 0 0.02 0.2 2

EphA7-112Kd

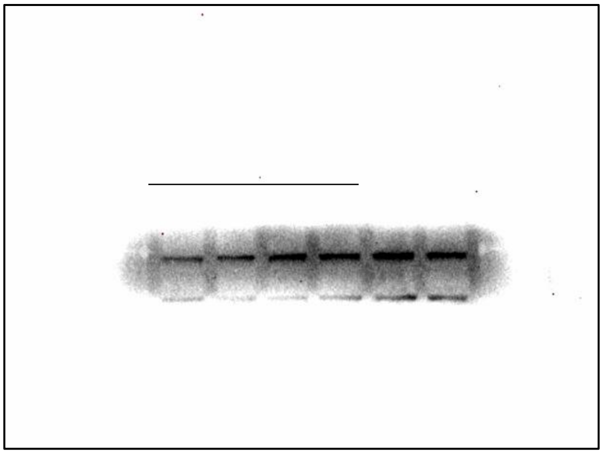

E2(ng/ml) 0 0.02 0.2 2

SP1-90Kd

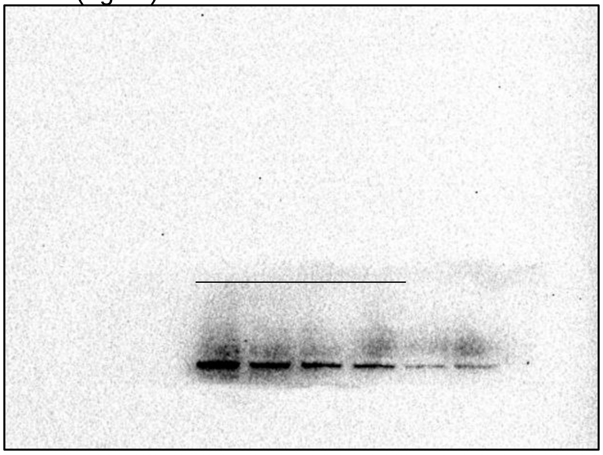

E2(ng/ml) 0 0.02 0.2 2

GAPDH-37Kd

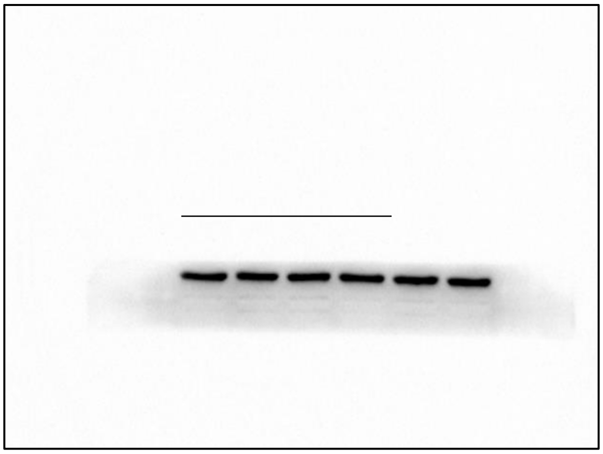

Fig.4D

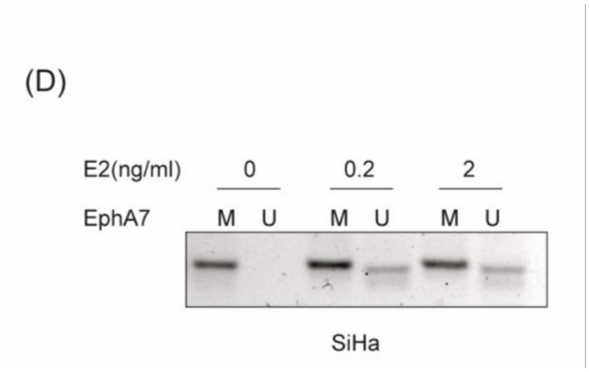

EphA7-MSP

M:229bp ;U:229bp

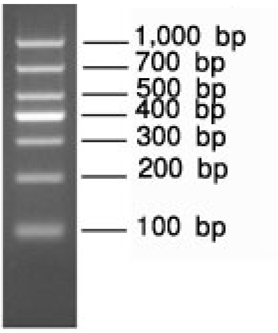

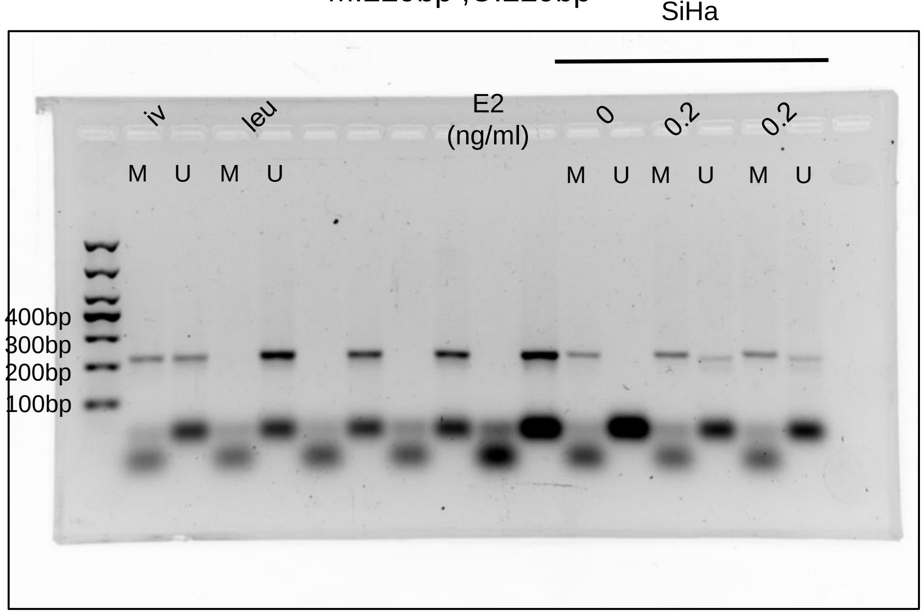

Supplement: Supplementary file 3 — Original data [file 41419_2025_7512_MOESM3_ESM.pdf]
